# Supplementary material for: Reduced miR-26b Expression in Megakaryocytes and Platelets Contributes to Elevated Level of Platelet Activation Status in Sepsis
Source: Int J Mol Sci. 2020 Jan 29;21(3):866. doi: 10.3390/ijms21030866 (PMC7036890; doi:10.3390/ijms21030866)
Supplement: Supplementary file 1 [file ijms-21-00866-s001.zip › ijms-696391-supp-final/Suppl.Table1_Szilagyi_final.docx]

**1. Down-regulated miRNAs:**

|  | **Assay ID and name** | **Rq value** |
| --- | --- | --- |
| **1.** | 002407_hsa-let-7e#_B | 0,070 |
| **2.** | 002391_hsa-miR-374b#_B | 0,086 |
| **3.** | 000508_hsa-miR-204_A | 0,099 |
| **4.** | 000480_hsa-miR-181a_A | 0,104 |
| **5.** | 002432_hsa-miR-625#_B | 0,105 |
| **6.** | 002263_hsa-miR-190b_B | 0,114 |
| **7.** | 002115_hsa-miR-26a-2#_B | 0,119 |
| **8.** | 002779_HSA-MIR-1271_B | 0,141 |
| **9.** | 000516_hsa-miR-213_B | 0,146 |
| **10.** | 001178_mmu-let-7d#_B | 0,157 |
| **11.** | 000457_hsa-miR-132_A | 0,158 |
| **12.** | 002096_hsa-miR-221#_B | 0,189 |
| **13.** | 000507_hsa-miR-203_A | 0,197 |
| **14.** | 002099_hsa-miR-224_A | 0,229 |
| **15.** | 000554_hsa-miR-361_A | 0,259 |
| **16.** | 002087_hsa-miR-505#_B | 0,259 |
| **17.** | 002433_hsa-miR-628-5p_A | 0,260 |
| **18.** | 000433_hsa-miR-95_A | 0,262 |
| **19.** | 002329_hsa-miR-452_A | 0,268 |
| **20.** | 002887_HSA-MIR-1250_B | 0,268 |
| **21.** | 001818_rno-miR-29c#_B | 0,269 |
| **22.** | 000471_hsa-miR-148b_A | 0,282 |
| **23.** | 002443_hsa-miR-26a-1#_B | 0,289 |
| **24.** | 002248_hsa-miR-142-5p_A | 0,308 |
| **25.** | 000512_hsa-miR-210_A | 0,311 |
| **26.** | 000460_hsa-miR-135a_A | 0,314 |
| **27.** | 002424_hsa-miR-19a#_B | 0,324 |
| **28.** | 001821_hsa-miR-484_A | 0,336 |
| **29.** | 002392_hsa-miR-301b_A | 0,336 |
| **30.** | 001585_hsa-miR-641_B | 0,341 |
| **31.** | 002333_hsa-miR-181c#_B | 0,341 |
| **32.** | 001518_hsa-miR-532_A | 0,343 |
| **33.** | 002827_HSA-MIR-1301_B | 0,346 |
| **34.** | 000419_hsa-miR-30c_A | 0,350 |
| **35.** | 002231_hsa-miR-9#_B | 0,353 |
| **36.** | 002444_hsa-miR-26b#_B | 0,365 |
| **37.** | 002185_hsa-miR-335#_B | 0,368 |
| **38.** | 002838_HSA-MIR-1291_B | 0,373 |
| **39.** | 002776_HSA-MIR-1179_B | 0,391 |
| **40.** | 002881_HSA-MIR-548E_B | 0,391 |
| **41.** | 000382_hsa-let-7f_A | 0,402 |
| **42.** | 002252_hsa-miR-338-3p_A | 0,402 |
| **43.** | 000510_hsa-miR-206_B | 0,410 |
| **44.** | 002434_hsa-miR-628-3p_B | 0,419 |
| **45.** | 002125_hsa-miR-374a#_B | 0,420 |
| **46.** | 002423_hsa-miR-18a#_B | 0,425 |
| **47.** | 002303_hsa-miR-450a_A | 0,427 |
| **48.** | 002425_hsa-miR-19b-1#_B | 0,429 |
| **49.** | 002237_hsa-miR-548d-5p_A | 0,433 |
| **50.** | 002258_hsa-miR-340_A | 0,433 |
| **51.** | 002314_hsa-miR-7-2#_B | 0,434 |
| **52.** | 002300_hsa-miR-200c_A | 0,455 |
| **53.** | 001043_hsa-miR-497_B | 0,458 |
| **54.** | 002897_HSA-MIR-664_B | 0,459 |
| **55.** | 001283_hsa-miR-363#_B | 0,460 |
| **56.** | 002409_hsa-miR-589_A | 0,463 |
| **57.** | 001319_mmu-miR-374-5p_A | 0,465 |
| **58.** | 002322_hsa-miR-671-3p_A | 0,465 |
| **59.** | 002160_hsa-miR-148b#_B | 0,466 |
| **60.** | 001605_hsa-miR-548d_A | 0,469 |
| **61.** | 001543_hsa-miR-589_B | 0,471 |
| **62.** | 002198_hsa-miR-125a-5p_A | 0,474 |
| **63.** | 002127_hsa-miR-376a#_B | 0,481 |
| **64.** | 001024_hsa-miR-429_A | 0,483 |
| **65.** | 002868_HSA-MIR-1249_B | 0,486 |
| **66.** | 002118_hsa-let-7g#_B | 0,490 |
| **67.** | 002429_hsa-miR-548c-5p_A | 0,501 |
| **68.** | 001998_hsa-miR-769-5p_B | 0,503 |
| **69.** | 001284_hsa-miR-542-3p_A | 0,504 |
| **70.** | 000602_hsa-miR-30b_A | 0,507 |
| **71.** | 002380_hsa-miR-106b#_B | 0,512 |
| **72.** | 002678_HSA-MIR-191#_B | 0,523 |
| **73.** | 000443_hsa-miR-107_A | 0,525 |
| **74.** | 002098_hsa-miR-223#_B | 0,525 |
| **75.** | 000268_dme-miR-7_B | 0,529 |
| **76.** | 002677_HSA-MIR-590-3P_B | 0,530 |
| **77.** | 000514_hsa-miR-211_A | 0,533 |
| **78.** | 001996_hsa-miR-454#_B | 0,534 |
| **79.** | 001020_hsa-miR-365_A | 0,540 |
| **80.** | 002251_hsa-miR-200b_A | 0,541 |
| **81.** | 001046_hsa-miR-500_B | 0,544 |
| **82.** | 002406_hsa-let-7e_A | 0,546 |
| **83.** | 002172_hsa-let-7i#_B | 0,546 |
| **84.** | 002309_hsa-miR-424#_B | 0,546 |
| **85.** | 000407_hsa-miR-26b_A | 0,547 |
| **86.** | 002173_hsa-miR-15b#_B | 0,548 |
| **87.** | 000543_hsa-miR-328_A | 0,549 |
| **88.** | 002312_hsa-miR-431#_B | 0,554 |
| **89.** | 001604_hsa-miR-651_A | 0,565 |
| **90.** | 002234_hsa-miR-140-3p_A | 0,566 |
| **91.** | 001986_hsa-miR-766_B | 0,567 |
| **92.** | 002642_HSA-MIR-151-5P_B | 0,568 |
| **93.** | 001562_hsa-miR-629_B | 0,569 |
| **94.** | 002325_hsa-miR-744#_B | 0,574 |
| **95.** | 002340_hsa-miR-423-5p_A | 0,579 |
| **96.** | 002117_hsa-miR-362-3p_A | 0,582 |
| **97.** | 002769_HSA-MIR-1227_B | 0,588 |
| **98.** | 002166_hsa-miR-29b-2#_B | 0,594 |
| **99.** | 000522_hsa-miR-219_A | 0,596 |
| **100.** | 002619_hsa-let-7b_A | 0,597 |
| **101.** | 002216_hsa-miR-128a_A | 0,598 |
| **102.** | 000473_hsa-miR-150_A | 0,602 |
| **103.** | 001090_mmu-miR-93_A | 0,602 |
| **104.** | 002301_hsa-miR-22#_B | 0,602 |
| **105.** | 002259_hsa-miR-340#_B | 0,604 |
| **106.** | 002276_hsa-miR-222_A | 0,609 |
| **107.** | 000515_hsa-miR-212_A | 0,612 |
| **108.** | 002148_hsa-miR-144#_B | 0,617 |
| **109.** | 002323_hsa-miR-454_A | 0,622 |
| **110.** | 002282_hsa-let-7g_A | 0,626 |
| **111.** | 000528_hsa-miR-301_A | 0,627 |
| **112.** | 002376_hsa-miR-543_B | 0,629 |
| **113.** | 002174_hsa-miR-27b#_B | 0,636 |
| **114.** | 001551_hsa-miR-597_A | 0,638 |
| **115.** | 002253_hsa-miR-101_A | 0,642 |
| **116.** | 000544_hsa-miR-330_A | 0,645 |
| **117.** | 002202_hsa-miR-889_A | 0,645 |
| **118.** | 002441_hsa-miR-24-2#_B | 0,655 |
| **119.** | 002302_hsa-miR-425#_B | 0,661 |
| **120.** | 002908_HSA-MIR-1296_B | 0,663 |
| **121.** | 000399_hsa-miR-23a_A | 0,665 |

**2. Upregulated miRNAs:**

|  | **Assay ID and name** | **Rq value** |
| --- | --- | --- |
| **1.** | 000572_hsa-miR-382_A | 1,506 |
| **2.** | 002279_hsa-miR-31_A | 1,508 |
| **3.** | 000435_hsa-miR-99a_A | 1,510 |
| **4.** | 000539_hsa-miR-324-5p_A | 1,517 |
| **5.** | 001558_hsa-miR-601_B | 1,549 |
| **6.** | 000565_hsa-miR-376a_A | 1,555 |
| **7.** | 000570_hsa-miR-380-5p_B | 1,557 |
| **8.** | 000402_hsa-miR-24_A | 1,571 |
| **9.** | 001138_mmu-miR-379_A | 1,580 |
| **10.** | 000379_hsa-let-7c_A | 1,619 |
| **11.** | 002364_hsa-miR-493_A | 1,623 |
| **12.** | 001973_U6 rRNA_A_B | 1,651 |
| **13.** | 000413_hsa-miR-29b_A | 1,654 |
| **14.** | 002414_hsa-miR-616_A | 1,669 |
| **15.** | 001611_hsa-miR-654_A | 1,682 |
| **16.** | 002844_HSA-MIR-320B_B | 1,688 |
| **17.** | 000478_hsa-miR-154#_B | 1,693 |
| **18.** | 002623_hsa-miR-155_A | 1,761 |
| **19.** | 002791_HSA-MIR-1244_B | 1,783 |
| **20.** | 001608_hsa-miR-449b_A | 1,831 |
| **21.** | 002188_hsa-miR-943_B | 1,881 |
| **22.** | 002275_hsa-miR-370_A | 1,894 |
| **23.** | 002277_hsa-miR-320_A | 1,926 |
| **24.** | 000426_hsa-miR-34a_A | 1,941 |
| **25.** | 002235_hsa-miR-509-5p_A | 2,013 |
| **26.** | 002313_hsa-miR-139-3p_A | 2,033 |
| **27.** | 001182_mmu-miR-124a_A | 2,082 |
| **28.** | 001036_hsa-miR-485-5p_A | 2,087 |
| **29.** | 002289_hsa-miR-139-5p_A | 2,102 |
| **30.** | 001568_hsa-miR-605_B | 2,145 |
| **31.** | 000452_hsa-miR-127_A | 2,188 |
| **32.** | 002249_hsa-miR-143_A | 2,210 |
| **33.** | 002265_hsa-miR-544_A | 2,278 |
| **34.** | 002332_hsa-miR-409-3p_B | 2,295 |
| **35.** | 002278_hsa-miR-145_A | 2,311 |
| **36.** | 001278_hsa-miR-486_A | 2,347 |
| **37.** | 002801_HSA-MIR-1255B_B | 2,427 |
| **38.** | 002093_hsa-miR-486-3p_A | 2,564 |
| **39.** | 000493_hsa-miR-194_A | 2,680 |
| **40.** | 001026_hsa-miR-432_B | 2,688 |
| **41.** | 000567_hsa-miR-378_B | 2,819 |
| **42.** | 002768_HSA-MIR-1233_B | 2,836 |
| **43.** | 001141_mmu-miR-451_A | 2,845 |
| **44.** | 002246_hsa-miR-133a_A | 2,917 |
| **45.** | 002367_hsa-miR-193b_A | 2,940 |
| **46.** | 000502_hsa-miR-200a_A | 2,968 |
| **47.** | 002895_HSA-MIR-720_B | 3,007 |
| **48.** | 000527_hsa-miR-296_A | 3,083 |
| **49.** | 002840_HSA-MIR-1275_B | 3,904 |
| **50.** | 002338_hsa-miR-483-5p_A | 3,907 |
| **51.** | 001512_hsa-miR-657_B | 4,156 |
| **52.** | 002884_HSA-MIR-1274B_B | 4,212 |
| **53.** | 000437_hsa-miR-100_A | 4,812 |
| **54.** | 001597_hsa-miR-645_B | 5,065 |
| **55.** | 002439_hsa-miR-23a#_B | 5,489 |
| **56.** | 002896_HSA-MIR-1260_B | 5,721 |
| **57.** | 002095_hsa-miR-219-1-3p_A | 5,734 |
| **58.** | 000186_mmu-miR-96_A | 7,155 |
| **59.** | 002883_HSA-MIR-1274A_B | 7,512 |
| **60.** | 001582_hsa-miR-638_B | 11,666 |
| **61.** | 002819_HSA-MIR-548K_B | 11,768 |

**3. Unchanged miRNAs:**

|  | **Assay ID and name** | **Rq value** |
| --- | --- | --- |
| **1.** | 000564_hsa-miR-375_A | 0,676 |
| **2.** | 001273_hsa-miR-362_A | 0,679 |
| **3.** | 002084_hsa-miR-504_A | 0,684 |
| **4.** | 002261_hsa-miR-135b_A | 0,685 |
| **5.** | 001021_hsa-miR-369-5p_A | 0,689 |
| **6.** | 002109_hsa-miR-32_A | 0,689 |
| **7.** | 000557_hsa-miR-369-3p_A | 0,690 |
| **8.** | 000422_hsa-miR-30e-3p_B | 0,693 |
| **9.** | 002230_hsa-miR-330-5p_A | 0,696 |
| **10.** | 002431_hsa-miR-625_A | 0,697 |
| **11.** | 002317_hsa-miR-181a-2#_B | 0,701 |
| **12.** | 000377_hsa-let-7a_A | 0,705 |
| **13.** | 000449_hsa-miR-125b_A | 0,706 |
| **14.** | 002212_hsa-miR-888_A | 0,708 |
| **15.** | 000409_hsa-miR-27b_A | 0,709 |
| **16.** | 000577_hsa-miR-98_A | 0,713 |
| **17.** | 001630_mmu-miR-491_A | 0,716 |
| **18.** | 001277_hsa-miR-485-3p_A | 0,720 |
| **19.** | 001663_mmu-miR-495_A | 0,722 |
| **20.** | 002233_hsa-miR-331-5p_A | 0,725 |
| **21.** | 000405_hsa-miR-26a_A | 0,727 |
| **22.** | 000468_hsa-miR-146a_A | 0,727 |
| **23.** | 000489_hsa-miR-190_A | 0,727 |
| **24.** | 002334_hsa-miR-182_A | 0,729 |
| **25.** | 001535_hsa-miR-551b_A | 0,730 |
| **26.** | 001510_hsa-miR-656_B | 0,731 |
| **27.** | 000546_hsa-miR-335_A | 0,734 |
| **28.** | 002239_hsa-miR-654-3p_A | 0,734 |
| **29.** | 002157_hsa-miR-337-3p_B | 0,740 |
| **30.** | 002196_hsa-miR-99b#_B | 0,744 |
| **31.** | 001014_hsa-miR-20b_A | 0,748 |
| **32.** | 001285_hsa-miR-487b_A | 0,753 |
| **33.** | 002255_hsa-miR-149_A | 0,753 |
| **34.** | 002850_HSA-MIR-1256_B | 0,754 |
| **35.** | 000397_hsa-miR-21_A | 0,755 |
| **36.** | 002437_hsa-miR-20a#_B | 0,756 |
| **37.** | 000395_hsa-miR-19a_A | 0,762 |
| **38.** | 000464_hsa-miR-142-3p_A | 0,762 |
| **39.** | 000497_hsa-miR-197_A | 0,767 |
| **40.** | 002305_hsa-miR-30d#_B | 0,769 |
| **41.** | 002285_hsa-miR-186_A | 0,771 |
| **42.** | 002143_hsa-miR-101#_B | 0,771 |
| **43.** | 002135_hsa-miR-33a_B | 0,781 |
| **44.** | 000533_hsa-miR-302c_A | 0,783 |
| **45.** | 001286_hsa-miR-539_A | 0,785 |
| **46.** | 002904_HSA-MIR-548L_B | 0,786 |
| **47.** | 001592_hsa-miR-642_A | 0,788 |
| **48.** | 001610_hsa-miR-411_A | 0,789 |
| **49.** | 002435_hsa-miR-501-3p_A | 0,793 |
| **50.** | 001514_hsa-miR-659_B | 0,798 |
| **51.** | 002355_hsa-miR-532-3p_A | 0,800 |
| **52.** | 000400_hsa-miR-23b_A | 0,804 |
| **53.** | 000524_hsa-miR-221_A | 0,805 |
| **54.** | 002283_hsa-let-7d_A | 0,805 |
| **55.** | 001591_hsa-miR-617_B | 0,805 |
| **56.** | 002792_HSA-MIR-1303_B | 0,806 |
| **57.** | 000500_hsa-miR-199b_A | 0,807 |
| **58.** | 002408_hsa-miR-548b-5p_A | 0,810 |
| **59.** | 000482_hsa-miR-181c_A | 0,813 |
| **60.** | 002083_hsa-miR-502-3p_A | 0,813 |
| **61.** | 000463_hsa-miR-141_A | 0,822 |
| **62.** | 002422_hsa-miR-18a_A | 0,822 |
| **63.** | 002215_hsa-miR-196b_A | 0,823 |
| **64.** | 002116_hsa-miR-361-3p_B | 0,823 |
| **65.** | 000390_hsa-miR-15b_A | 0,825 |
| **66.** | 000604_hsa-miR-424_A | 0,829 |
| **67.** | 002361_hsa-miR-146b-3p_A | 0,839 |
| **68.** | 002260_hsa-miR-342-3p_A | 0,843 |
| **69.** | 002446_hsa-miR-28-3p_A | 0,847 |
| **70.** | 002266_hsa-miR-545#_B | 0,847 |
| **71.** | 000431_hsa-miR-92a_A | 0,848 |
| **72.** | 002003_hsa-miR-769-3p_B | 0,852 |
| **73.** | 002420_hsa-miR-16-1#_B | 0,854 |
| **74.** | 000420_hsa-miR-30d_B | 0,855 |
| **75.** | 002284_hsa-miR-138_A | 0,857 |
| **76.** | 000411_hsa-miR-28_A | 0,861 |
| **77.** | 000494_hsa-miR-195_A | 0,861 |
| **78.** | 002398_hsa-miR-579_A | 0,862 |
| **79.** | 002445_hsa-miR-27a#_B | 0,863 |
| **80.** | 002386_hsa-miR-523_A | 0,865 |
| **81.** | 002112_hsa-miR-29a_A | 0,869 |
| **82.** | 002106_hsa-miR-188-3p_A | 0,872 |
| **83.** | 002351_hsa-miR-576-3p_A | 0,876 |
| **84.** | 002238_hsa-miR-411#_B | 0,885 |
| **85.** | 002204_hsa-miR-875-3p_A | 0,888 |
| **86.** | 001593_hsa-miR-618_A | 0,889 |
| **87.** | 002182_hsa-miR-939_B | 0,897 |
| **88.** | 002907_HSA-MIR-1825_B | 0,899 |
| **89.** | 001109_hsa-miR-502_A | 0,904 |
| **90.** | 002324_hsa-miR-744_A | 0,905 |
| **91.** | 001990_hsa-miR-758_A | 0,906 |
| **92.** | 002187_hsa-miR-942_B | 0,906 |
| **93.** | 002789_HSA-MIR-1269_B | 0,909 |
| **94.** | 001187_mmu-miR-140_A | 0,914 |
| **95.** | 002102_hsa-miR-34b_B | 0,924 |
| **96.** | 002346_hsa-miR-551b#_B | 0,925 |
| **97.** | 000439_hsa-miR-103_A | 0,927 |
| **98.** | 001338_rno-miR-7#_B | 0,935 |
| **99.** | 002331_hsa-miR-409-5p_A | 0,939 |
| **100.** | 001584_hsa-miR-640_B | 0,939 |
| **101.** | 001186_mmu-miR-134_A | 0,940 |
| **102.** | 000436_hsa-miR-99b_A | 0,946 |
| **103.** | 001028_hsa-miR-433_A | 0,949 |
| **104.** | 001120_hsa-miR-520f_A | 0,957 |
| **105.** | 002447_hsa-miR-29a#_B | 0,957 |
| **106.** | 002418_hsa-let-7f-2#_B | 0,962 |
| **107.** | 000583_hsa-miR-9_A | 0,964 |
| **108.** | 000387_hsa-miR-10a_A | 0,969 |
| **109.** | 002428_hsa-miR-500_A | 0,972 |
| **110.** | 000442_hsa-miR-106b_A | 0,974 |
| **111.** | 001102_hsa-miR-376b_A | 0,974 |
| **112.** | 002352_hsa-miR-652_A | 0,981 |
| **113.** | 001557_hsa-miR-624_B | 0,981 |
| **114.** | 001048_hsa-miR-503_A | 0,982 |
| **115.** | 002128_hsa-miR-377#_B | 0,983 |
| **116.** | 002184_hsa-miR-339-3p_A | 0,990 |
| **117.** | 001094_RNU44_A_B | 0,995 |
| **118.** | 000391_hsa-miR-16_A | 0,997 |
| **119.** | 002438_hsa-miR-21#_B | 1,000 |
| **120.** | 002316_hsa-miR-34a#_B | 1,000 |
| **121.** | 002315_hsa-miR-10b#_B | 1,000 |
| **122.** | 002311_hsa-miR-20b#_B | 1,000 |
| **123.** | 002002_hsa-miR-770-5p_B | 1,000 |
| **124.** | 001823_hsa-miR-512-3p_A | 1,000 |
| **125.** | 000580_hsa-miR-20a_A | 1,004 |
| **126.** | 000456_hsa-miR-130b_A | 1,005 |
| **127.** | 002229_hsa-miR-127-5p_A | 1,009 |
| **128.** | 002358_hsa-miR-489_A | 1,014 |
| **129.** | 001101_hsa-miR-329_A | 1,015 |
| **130.** | 001097_hsa-miR-146b_A | 1,019 |
| **131.** | 002766_HSA-MIR-1225-3P_B | 1,022 |
| **132.** | 002164_hsa-miR-149#_B | 1,025 |
| **133.** | 001601_hsa-miR-648_B | 1,027 |
| **134.** | 001984_hsa-miR-590-5p_A | 1,037 |
| **135.** | 001052_hsa-miR-508_A | 1,038 |
| **136.** | 002436_hsa-miR-629_A | 1,038 |
| **137.** | 001271_hsa-miR-363_A | 1,046 |
| **138.** | 002783_HSA-MIR-548J_B | 1,050 |
| **139.** | 000398_hsa-miR-22_A | 1,055 |
| **140.** | 000563_hsa-miR-374_A | 1,055 |
| **141.** | 002296_hsa-miR-885-5p_A | 1,057 |
| **142.** | 001615_hsa-miR-573_B | 1,063 |
| **143.** | 001992_hsa-miR-668_B | 1,064 |
| **144.** | 002136_hsa-miR-33a#_B | 1,065 |
| **145.** | 002308_hsa-miR-17_A | 1,071 |
| **146.** | 000403_hsa-miR-25_A | 1,073 |
| **147.** | 001988_hsa-miR-598_A | 1,075 |
| **148.** | 000389_hsa-miR-15a_A | 1,079 |
| **149.** | 000491_hsa-miR-192_A | 1,086 |
| **150.** | 000417_hsa-miR-30a-5p_B | 1,086 |
| **151.** | 002089_hsa-miR-505_A | 1,087 |
| **152.** | 002100_hsa-miR-136#_B | 1,097 |
| **153.** | 000451_hsa-miR-126#_B | 1,103 |
| **154.** | 002139_hsa-miR-93#_B | 1,104 |
| **155.** | 000542_hsa-miR-326_A | 1,116 |
| **156.** | 000498_hsa-miR-199a_A | 1,121 |
| **157.** | 002304_hsa-miR-199a-3p_A | 1,132 |
| **158.** | 001193_mmu-miR-187_A | 1,142 |
| **159.** | 002257_hsa-miR-339-5p_A | 1,144 |
| **160.** | 000416_hsa-miR-30a-3p_B | 1,149 |
| **161.** | 002254_hsa-miR-151-3p_B | 1,154 |
| **162.** | 002271_hsa-miR-185_A | 1,155 |
| **163.** | 001516_hsa-miR-425-5p_A | 1,169 |
| **164.** | 001515_hsa-miR-660_A | 1,179 |
| **165.** | 002847_HSA-MIR-1180_B | 1,179 |
| **166.** | 002169_hsa-miR-106a_A | 1,183 |
| **167.** | 002183_hsa-miR-941_B | 1,184 |
| **168.** | 000529_hsa-miR-302a_A | 1,198 |
| **169.** | 001030_hsa-miR-449_A | 1,212 |
| **170.** | 000587_hsa-miR-29c_A | 1,213 |
| **171.** | 002186_hsa-miR-345_A | 1,226 |
| **172.** | 002193_hsa-miR-886-5p_A | 1,229 |
| **173.** | 000521_hsa-miR-218_A | 1,247 |
| **174.** | 002816_HSA-MIR-548H_B | 1,252 |
| **175.** | 002122_hsa-miR-376c_A | 1,261 |
| **176.** | 002108_hsa-miR-30c-1#_B | 1,268 |
| **177.** | 001624_hsa-miR-584_B | 1,271 |
| **178.** | 001979_hsa-miR-431_A | 1,277 |
| **179.** | 002365_hsa-miR-494_A | 1,289 |
| **180.** | 002207_hsa-miR-450b-5p_A | 1,291 |
| **181.** | 002675_HSA-MIR-577_B | 1,291 |
| **182.** | 000477_hsa-miR-154_A | 1,318 |
| **183.** | 002149_hsa-miR-145#_B | 1,321 |
| **184.** | 002415_hsa-miR-519a_A | 1,334 |
| **185.** | 002281_hsa-miR-193a-5p_A | 1,344 |
| **186.** | 000518_hsa-miR-215_A | 1,349 |
| **187.** | 000470_hsa-miR-148a_A | 1,350 |
| **188.** | 000545_hsa-miR-331_A | 1,352 |
| **189.** | 000571_hsa-miR-381_A | 1,354 |
| **190.** | 002264_hsa-miR-872_A | 1,354 |
| **191.** | 002272_hsa-miR-192#_B | 1,355 |
| **192.** | 002161_hsa-miR-324-3p_A | 1,357 |
| **193.** | 002247_hsa-miR-133b_A | 1,358 |
| **194.** | 000475_hsa-miR-152_A | 1,362 |
| **195.** | 002341_hsa-miR-708_A | 1,363 |
| **196.** | 001274_hsa-miR-410_A | 1,377 |
| **197.** | 002137_hsa-miR-92a-1#_B | 1,384 |
| **198.** | 002228_hsa-miR-126_A | 1,394 |
| **199.** | 002270_hsa-miR-183#_B | 1,414 |
| **200.** | 000454_hsa-miR-130a_A | 1,439 |
| **201.** | 002318_hsa-miR-453_A | 1,440 |
| **202.** | 002156_hsa-miR-337-5p_A | 1,455 |
| **203.** | 002410_hsa-miR-550_B | 1,455 |
| **204.** | 002194_hsa-miR-886-3p_A | 1,461 |
| **205.** | 002818_HSA-MIR-1254_B | 1,461 |
| **206.** | 002299_hsa-miR-191_A | 1,471 |
| **207.** | 002295_hsa-miR-223_A | 1,474 |
| **208.** | 002297_hsa-miR-422a_A | 1,484 |

**Suppl. Table 1.**
