# Supplementary material for: Reduced miR-26b Expression in Megakaryocytes and Platelets Contributes to Elevated Level of Platelet Activation Status in Sepsis
Source: Int J Mol Sci. 2020 Jan 29;21(3):866. doi: 10.3390/ijms21030866 (PMC7036890; doi:10.3390/ijms21030866)
Supplement: Supplementary file 1 [file ijms-21-00866-s001.zip › ijms-696391-supp-final/Suppl.Table2_Szilagyi_final.docx]

| **Gene ID** | **P-value** | **Regulation ([LPS] vs. [CTRL])** | **FC ([LPS] vs. [CTRL])** | **Gene Symbol** |
| --- | --- | --- | --- | --- |
| ENSG00000154997 | 0.00261 | up | 1.6885909 | *SEPT14* |
| ENSG00000149313 | 0.00277 | up | 1.5268371 | *AASDHPPT* |
| ENSG00000111644 | 0.00038 | up | 1.664306 | *ACRBP* |
| ENSG00000005187 | 0.00146 | up | 1.5425946 | *ACSM3* |
| ENSG00000008277 | 0.00649 | up | 1.5186503 | *ADAM22* |
| ENSG00000214273 | 0.02118 | up | 1.9641005 | *AGGF1P1* |
| ENSG00000233435 | 0.00223 | up | 2.8668454 | *AGGF1P2* |
| ENSG00000177674 | 0.00011 | up | 3.910572 | *AGTRAP* |
| ENSG00000204472 | 0.00109 | up | 2.0455136 | *AIF1* |
| ENSG00000004455 | 0.00000 | up | 1.5164824 | *AK2* |
| ENSG00000158578 | 0.00022 | up | 2.424636 | *ALAS2* |
| ENSG00000159423 | 0.00041 | up | 1.5291283 | *ALDH4A1* |
| ENSG00000164904 | 0.00632 | up | 1.7676355 | *ALDH7A1* |
| ENSG00000230002 | 0.01462 | up | 2.0018396 | *ALMS1-IT1* |
| ENSG00000230453 | 0.01174 | up | 1.6666868 | *ANKRD18B* |
| ENSG00000105186 | 0.00008 | up | 1.7380158 | *ANKRD27* |
| ENSG00000077420 | 0.00164 | up | 1.5729474 | *APBB1IP* |
| ENSG00000179750 | 0.01525 | up | 1.5900651 | *APOBEC3B* |
| ENSG00000167580 | 0.01788 | up | 1.5530784 | *AQP2* |
| ENSG00000120318 | 0.00007 | up | 1.5528275 | *ARAP3* |
| ENSG00000142632 | 0.02815 | up | 1.5344329 | *ARHGEF19* |
| ENSG00000173409 | 0.00026 | up | 1.6807091 | *ARV1* |
| ENSG00000072182 | 0.00087 | up | 1.8053335 | *ASIC4* |
| ENSG00000138363 | 0.00009 | up | 1.5006775 | *ATIC* |
| ENSG00000241837 | 0.00094 | up | 1.5690398 | *ATP5O* |
| ENSG00000123191 | 0.01472 | up | 1.5152377 | *ATP7B* |
| ENSG00000167601 | 0.00007 | up | 1.921462 | *AXL* |
| ENSG00000198604 | 0.00058 | up | 1.5185059 | *BAZ1A* |
| ENSG00000140379 | 0.00002 | up | 1.636915 | *BCL2A1* |
| ENSG00000110987 | 0.01229 | up | 1.5382286 | *BCL7A* |
| ENSG00000236824 | 0.01375 | up | 1.6843061 | *BCYRN1* |
| ENSG00000023445 | 0.00001 | up | 2.8367584 | *BIRC3* |
| ENSG00000174808 | 0.01216 | up | 1.9281745 | *BTC* |
| ENSG00000150076 | 0.03551 | up | 2.5714703 | *C10orf68* |
| ENSG00000224877 | 0.01707 | up | 1.5438092 | *C17orf89* |
| ENSG00000249087 | 0.00293 | up | 1.9556997 | *C1orf213* |
| ENSG00000256073 | 0.00299 | up | 1.8287226 | *C21orf119* |
| ENSG00000186132 | 0.00074 | up | 1.8429319 | *C2orf76* |
| ENSG00000182600 | 0.02939 | up | 1.7491248 | *C2orf82* |
| ENSG00000171860 | 0.01040 | up | 1.8114684 | *C3AR1* |
| ENSG00000174899 | 0.00230 | up | 1.8838063 | *C3orf55* |
| ENSG00000104327 | 0.00036 | up | 1.9176925 | *CALB1* |
| ENSG00000105974 | 0.00620 | up | 1.7824409 | *CAV1* |
| ENSG00000105971 | 0.00073 | up | 1.7130595 | *CAV2* |
| ENSG00000159228 | 0.02738 | up | 1.5338968 | *CBR1* |
| ENSG00000242715 | 0.01714 | up | 1.672615 | *CCDC169* |
| ENSG00000151838 | 0.00990 | up | 2.4374661 | *CCDC175* |
| ENSG00000213085 | 0.00002 | up | 1.6527905 | *CCDC19* |
| ENSG00000108691 | 0.00000 | up | 1.6228354 | *CCL2* |
| ENSG00000129277 | 0.00009 | up | 2.3087227 | *CCL4* |
| ENSG00000112486 | 0.00054 | up | 3.1725464 | *CCR6* |
| ENSG00000126353 | 0.00094 | up | 1.5655668 | *CCR7* |
| ENSG00000163606 | 0.03887 | up | 2.4213512 | *CD200R1* |
| ENSG00000101017 | 0.00010 | up | 1.6167153 | *CD40* |
| ENSG00000244703 | 0.04478 | up | 1.8334956 | *CD46P1* |
| ENSG00000196776 | 0.00019 | up | 1.5098263 | *CD47* |
| ENSG00000085117 | 0.00001 | up | 1.7457954 | *CD82* |
| ENSG00000164885 | 0.01679 | up | 1.6738747 | *CDK5* |
| ENSG00000123080 | 0.00034 | up | 1.9618324 | *CDKN2C* |
| ENSG00000090661 | 0.00816 | up | 1.9767569 | *CERS4* |
| ENSG00000143942 | 0.01072 | up | 1.6275241 | *CHAC2* |
| ENSG00000263846 | 0.02402 | up | 1.9405805 | *CIAPIN1P* |
| ENSG00000122873 | 0.01053 | up | 1.5410776 | *CISD1* |
| ENSG00000105472 | 0.00007 | up | 3.1845932 | *CLEC11A* |
| ENSG00000159212 | 0.00013 | up | 1.9478717 | *CLIC6* |
| ENSG00000128973 | 0.00199 | up | 1.5127425 | *CLN6* |
| ENSG00000119946 | 0.02389 | up | 1.7517104 | *CNNM1* |
| ENSG00000257727 | 0.00481 | up | 1.5269316 | *CNPY2* |
| ENSG00000182871 | 0.00004 | up | 1.6476551 | *COL18A1* |
| ENSG00000050767 | 0.00006 | up | 1.7108688 | *COL23A1* |
| ENSG00000163751 | 0.00002 | up | 2.5390666 | *CPA3* |
| ENSG00000088766 | 0.00001 | up | 1.50551 | *CRLS1* |
| ENSG00000184371 | 0.00001 | up | 1.7142476 | *CSF1* |
| ENSG00000077984 | 0.00159 | up | 1.6434329 | *CST7* |
| ENSG00000103811 | 0.00053 | up | 1.6006474 | *CTSH* |
| ENSG00000226950 | 0.00102 | up | 1.5783879 | *DANCR* |
| ENSG00000128185 | 0.01945 | up | 1.6065745 | *DGCR6L* |
| ENSG00000228002 | 0.01588 | up | 1.5267744 | *DHX9P1* |
| ENSG00000235706 | 0.02240 | up | 1.5737609 | *DICER1-AS1* |
| ENSG00000105612 | 0.04322 | up | 1.6529927 | *DNASE2* |
| ENSG00000166171 | 0.04811 | up | 1.682706 | *DPCD* |
| ENSG00000134146 | 0.02361 | up | 1.5503645 | *DPH6* |
| ENSG00000162999 | 0.00418 | up | 1.5949354 | *DUSP19* |
| ENSG00000158716 | 0.03123 | up | 1.506202 | *DUSP23* |
| ENSG00000108861 | 0.00013 | up | 1.571344 | *DUSP3* |
| ENSG00000198721 | 0.00079 | up | 1.7742213 | *ECI2* |
| ENSG00000136160 | 0.00014 | up | 1.5750343 | *EDNRB* |
| ENSG00000115380 | 0.02513 | up | 2.4602487 | *EFEMP1* |
| ENSG00000070785 | 0.00062 | up | 1.5036376 | *EIF2B3* |
| ENSG00000151023 | 0.00804 | up | 2.499297 | *ENKUR* |
| ENSG00000166947 | 0.00048 | up | 3.0908587 | *EPB42* |
| ENSG00000154928 | 0.00008 | up | 2.4492054 | *EPHB1* |
| ENSG00000196411 | 0.00030 | up | 1.7095072 | *EPHB4* |
| ENSG00000229007 | 0.02245 | up | 1.599151 | *EXOSC3P1* |
| ENSG00000121104 | 0.00004 | up | 1.5656765 | *FAM117A* |
| ENSG00000237765 | 0.00077 | up | 1.5186908 | *FAM200B* |
| ENSG00000237198 | 0.00069 | up | 3.098507 | *FAM27E1* |
| ENSG00000204805 | 0.01137 | up | 1.7468708 | *FAM27E4* |
| ENSG00000118894 | 0.04264 | up | 1.5481685 | *FAM86A* |
| ENSG00000119812 | 0.00307 | up | 1.5059526 | *FAM98A* |
| ENSG00000161040 | 0.00022 | up | 1.8483896 | *FBXL13* |
| ENSG00000179639 | 0.00011 | up | 2.4192972 | *FCER1A* |
| ENSG00000158869 | 0.00047 | up | 1.7094604 | *FCER1G* |
| ENSG00000103241 | 0.03532 | up | 2.2212083 | *FOXF1* |
| ENSG00000187474 | 0.01447 | up | 2.0573354 | *FPR3* |
| ENSG00000104290 | 0.01861 | up | 1.6036171 | *FZD3* |
| ENSG00000174804 | 0.01313 | up | 1.5977253 | *FZD4* |
| ENSG00000102287 | 0.00049 | up | 1.6293693 | *GABRE* |
| ENSG00000128683 | 0.00171 | up | 1.808596 | *GAD1* |
| ENSG00000136895 | 0.04217 | up | 1.7293805 | *GARNL3* |
| ENSG00000117226 | 0.00028 | up | 1.6069345 | *GBP3* |
| ENSG00000213512 | 0.00122 | up | 1.8560271 | *GBP7* |
| ENSG00000135414 | 0.00001 | up | 2.0822818 | *GDF11* |
| ENSG00000152147 | 0.00022 | up | 1.6147232 | *GEMIN6* |
| ENSG00000134864 | 0.00320 | up | 1.5375246 | *GGACT* |
| ENSG00000204136 | 0.01345 | up | 2.6734798 | *GGTA1P* |
| ENSG00000131153 | 0.00694 | up | 1.5980192 | *GINS2* |
| ENSG00000137198 | 0.00181 | up | 1.5250547 | *GMPR* |
| ENSG00000088053 | 0.00000 | up | 2.5472693 | *GP6* |
| ENSG00000164604 | 0.00021 | up | 2.2418485 | *GPR85* |
| ENSG00000152822 | 0.01701 | up | 3.1819007 | *GRM1* |
| ENSG00000084207 | 0.00004 | up | 2.4609137 | *GSTP1* |
| ENSG00000170180 | 0.00004 | up | 2.608954 | *GYPA* |
| ENSG00000250361 | 0.00100 | up | 2.718675 | *GYPB* |
| ENSG00000197465 | 0.00002 | up | 1.5820493 | *GYPE* |
| ENSG00000111713 | 0.00418 | up | 3.458254 | *GYS2* |
| ENSG00000145649 | 0.00016 | up | 4.66571 | *GZMA* |
| ENSG00000099284 | 0.04298 | up | 1.5647688 | *H2AFY2* |
| ENSG00000213934 | 0.00038 | up | 2.2331226 | *HBG1* |
| ENSG00000196565 | 0.00048 | up | 2.2382329 | *HBG2* |
| ENSG00000198130 | 0.00034 | up | 1.5976099 | *HIBCH* |
| ENSG00000214922 | 0.00609 | up | 2.1284838 | *HLA-F-AS1* |
| ENSG00000124097 | 0.02420 | up | 1.5605897 | *HMGB1P1* |
| ENSG00000254982 | 0.00541 | up | 1.5267917 | *HMGB1P24* |
| ENSG00000213467 | 0.01546 | up | 1.5206424 | *HMGB1P37* |
| ENSG00000226977 | 0.00000 | up | 1.9971586 | *HMGN1P24* |
| ENSG00000179362 | 0.00947 | up | 1.6624905 | *HMGN2P46* |
| ENSG00000150540 | 0.01291 | up | 3.775925 | *HNMT* |
| ENSG00000259051 | 0.02499 | up | 3.049053 | *HNRNPUP1* |
| ENSG00000215271 | 0.00022 | up | 1.8626423 | *HOMEZ* |
| ENSG00000186603 | 0.01856 | up | 1.8768231 | *HPDL* |
| ENSG00000173083 | 0.00856 | up | 1.5479423 | *HPSE* |
| ENSG00000119471 | 0.00613 | up | 1.5750878 | *HSDL2* |
| ENSG00000205100 | 0.01981 | up | 1.7525157 | *HSP90AA4P* |
| ENSG00000213430 | 0.00246 | up | 1.6399585 | *HSPD1P1* |
| ENSG00000115541 | 0.01449 | up | 1.5731807 | *HSPE1* |
| ENSG00000137965 | 0.00543 | up | 1.5238779 | *IFI44* |
| ENSG00000137959 | 0.00097 | up | 1.8540957 | *IFI44L* |
| ENSG00000126709 | 0.00009 | up | 2.089669 | *IFI6* |
| ENSG00000185745 | 0.00046 | up | 1.6111734 | *IFIT1* |
| ENSG00000152778 | 0.00158 | up | 1.7410975 | *IFIT5* |
| ENSG00000159217 | 0.00970 | up | 1.6866513 | *IGF2BP1* |
| ENSG00000115457 | 0.02092 | up | 1.5430794 | *IGFBP2* |
| ENSG00000115461 | 0.00076 | up | 1.6150508 | *IGFBP5* |
| ENSG00000163702 | 0.01277 | up | 1.5310626 | *IL17RC* |
| ENSG00000125538 | 0.01253 | up | 1.614417 | *IL1B* |
| ENSG00000104432 | 0.02332 | up | 2.1082993 | *IL7* |
| ENSG00000259673 | 0.02273 | up | 2.1143901 | *IQCH-AS1* |
| ENSG00000187608 | 0.00011 | up | 1.6211516 | *ISG15* |
| ENSG00000214401 | 0.00102 | up | 2.196202 | *KANSL1-AS1* |
| ENSG00000189337 | 0.00054 | up | 1.6643512 | *KAZN* |
| ENSG00000165572 | 0.01192 | up | 1.6478623 | *KBTBD6* |
| ENSG00000124780 | 0.00624 | up | 2.83433 | *KCNK17* |
| ENSG00000128052 | 0.00023 | up | 1.7634404 | *KDR* |
| ENSG00000258790 | 0.00664 | up | 1.549451 | *KIAA0391* |
| ENSG00000105610 | 0.00005 | up | 3.006215 | *KLF1* |
| ENSG00000185909 | 0.00551 | up | 1.9231862 | *KLHDC8B* |
| ENSG00000126790 | 0.02334 | up | 1.7019236 | *L3HYPDH* |
| ENSG00000179630 | 0.00063 | up | 1.702778 | *LACC1* |
| ENSG00000259974 | 0.00058 | up | 1.6821685 | *LINC00261* |
| ENSG00000258791 | 0.00049 | up | 2.2727842 | *LINC00520* |
| ENSG00000266904 | 0.00154 | up | 4.4539824 | *LINC00663* |
| ENSG00000250334 | 0.02726 | up | 4.176035 | *LINC00989* |
| ENSG00000250337 | 0.01105 | up | 1.7451222 | *LINC01021* |
| ENSG00000135363 | 0.00000 | up | 1.8727794 | *LMO2* |
| ENSG00000048540 | 0.00070 | up | 2.2150686 | *LMO3* |
| ENSG00000204482 | 0.00119 | up | 2.0781598 | *LST1* |
| ENSG00000049323 | 0.00048 | up | 1.5437591 | *LTBP1* |
| ENSG00000145220 | 0.00585 | up | 1.5127147 | *LYAR* |
| ENSG00000133800 | 0.00071 | up | 2.1552703 | *LYVE1* |
| ENSG00000261366 | 0.04916 | up | 3.2859461 | *MANEA-AS1* |
| ENSG00000184368 | 0.00089 | up | 2.0359678 | *MAP7D2* |
| ENSG00000247626 | 0.02014 | up | 1.530374 | *MARS2* |
| ENSG00000155254 | 0.00015 | up | 2.0267556 | *MARVELD1* |
| ENSG00000065328 | 0.00443 | up | 1.5280901 | *MCM10* |
| ENSG00000175471 | 0.00064 | up | 1.6982684 | *MCTP1* |
| ENSG00000214548 | 0.00000 | up | 2.1698096 | *MEG3* |
| ENSG00000123427 | 0.00190 | up | 1.6428783 | *METTL21B* |
| ENSG00000138382 | 0.00115 | up | 1.6087734 | *METTL5* |
| ENSG00000128268 | 0.00017 | up | 1.529933 | *MGAT3* |
| ENSG00000085871 | 0.00535 | up | 1.5161663 | *MGST2* |
| ENSG00000273340 | 0.03580 | up | 3.8159502 | *MICE* |
| ENSG00000253522 | 0.00000 | up | 1.8383592 | *MIR146A* |
| ENSG00000234883 | 0.00002 | up | 2.4564016 | *MIR155HG* |
| ENSG00000207939 | 0.00244 | up | 1.6708276 | *MIR223* |
| ENSG00000199133 | 0.02350 | up | 2.4891026 | *MIRLET7D* |
| ENSG00000132763 | 0.00226 | up | 1.5183659 | *MMACHC* |
| ENSG00000087245 | 0.00008 | up | 1.7735124 | *MMP2* |
| ENSG00000138722 | 0.02739 | up | 2.5541837 | *MMRN1* |
| ENSG00000173141 | 0.00094 | up | 1.5588231 | *MRP63* |
| ENSG00000262814 | 0.02935 | up | 2.051172 | *MRPL12* |
| ENSG00000226253 | 0.02180 | up | 2.0847673 | *MRPL35P3* |
| ENSG00000239789 | 0.01833 | up | 1.5009844 | *MRPS17* |
| ENSG00000120832 | 0.00537 | up | 1.5685571 | *MTERFD3* |
| ENSG00000157601 | 0.00023 | up | 4.57175 | *MX1* |
| ENSG00000214114 | 0.00017 | up | 1.6025387 | *MYCBP* |
| ENSG00000134323 | 0.00018 | up | 3.0921938 | *MYCN* |
| ENSG00000120279 | 0.00290 | up | 1.6783487 | *MYCT1* |
| ENSG00000198336 | 0.00013 | up | 2.9049463 | *MYL4* |
| ENSG00000150456 | 0.01621 | up | 1.6957793 | *N6AMT2* |
| ENSG00000119013 | 0.01329 | up | 1.5974898 | *NDUFB3* |
| ENSG00000168653 | 0.01327 | up | 1.5200485 | *NDUFS5* |
| ENSG00000160194 | 0.00086 | up | 1.5382715 | *NDUFV3* |
| ENSG00000100285 | 0.00004 | up | 2.0907176 | *NEFH* |
| ENSG00000136098 | 0.00634 | up | 1.5135592 | *NEK3* |
| ENSG00000184613 | 0.00407 | up | 1.5154244 | *NELL2* |
| ENSG00000123405 | 0.01080 | up | 1.6539168 | *NFE2* |
| ENSG00000230257 | 0.00085 | up | 2.5071821 | *NFE4* |
| ENSG00000187566 | 0.01586 | up | 1.6001142 | *NHLRC1* |
| ENSG00000163072 | 0.00101 | up | 2.5710566 | *NOSTRIN* |
| ENSG00000113389 | 0.01027 | up | 2.1990921 | *NPR3* |
| ENSG00000175745 | 0.00488 | up | 1.5973493 | *NR2F1* |
| ENSG00000135318 | 0.00400 | up | 1.894198 | *NT5E* |
| ENSG00000133636 | 0.00002 | up | 1.9652233 | *NTS* |
| ENSG00000143552 | 0.00000 | up | 1.8328431 | *NUP210L* |
| ENSG00000021762 | 0.00043 | up | 1.53306 | *OSBPL5* |
| ENSG00000254535 | 0.02132 | up | 1.7672985 | *PABPC4L* |
| ENSG00000254244 | 0.00398 | up | 1.5637758 | *PAICSP4* |
| ENSG00000273344 | 0.01087 | up | 1.8167348 | *PAXIP1-AS1* |
| ENSG00000150275 | 0.00000 | up | 1.6280326 | *PCDH15* |
| ENSG00000169174 | 0.01136 | up | 3.096832 | *PCSK9* |
| ENSG00000102230 | 0.00091 | up | 1.9779826 | *PCYT1B* |
| ENSG00000154678 | 0.02578 | up | 1.5682138 | *PDE1C* |
| ENSG00000229332 | 0.01051 | up | 1.8664094 | *PGBD4P8* |
| ENSG00000008438 | 0.01246 | up | 2.6948886 | *PGLYRP1* |
| ENSG00000043143 | 0.00001 | up | 1.5803188 | *PHF15* |
| ENSG00000142892 | 0.01293 | up | 1.5584736 | *PIGK* |
| ENSG00000060642 | 0.00008 | up | 1.7968181 | *PIGV* |
| ENSG00000141506 | 0.00739 | up | 2.3917763 | *PIK3R5* |
| ENSG00000101333 | 0.00729 | up | 2.8608065 | *PLCB4* |
| ENSG00000115896 | 0.00801 | up | 1.852296 | *PLCL1* |
| ENSG00000179598 | 0.02366 | up | 1.9701852 | *PLD6* |
| ENSG00000126822 | 0.00001 | up | 2.0567856 | *PLEKHG3* |
| ENSG00000130300 | 0.00007 | up | 1.823549 | *PLVAP* |
| ENSG00000120594 | 0.00006 | up | 1.5917414 | *PLXDC2* |
| ENSG00000221866 | 0.00036 | up | 1.6712857 | *PLXNA4* |
| ENSG00000161980 | 0.01006 | up | 1.6107174 | *POLR3K* |
| ENSG00000205808 | 0.01550 | up | 1.5610805 | *PPAPDC2* |
| ENSG00000168938 | 0.00041 | up | 1.8011659 | *PPIC* |
| ENSG00000197870 | 0.02334 | up | 1.8118659 | *PRB3* |
| ENSG00000186652 | 0.01209 | up | 1.7822238 | *PRG2* |
| ENSG00000186654 | 0.00005 | up | 1.6031531 | *PRR5* |
| ENSG00000099256 | 0.03992 | up | 1.5221664 | *PRTFDC1* |
| ENSG00000160013 | 0.00000 | up | 1.5967734 | *PTGIR* |
| ENSG00000101417 | 0.03725 | up | 1.7374692 | *PXMP4* |
| ENSG00000145337 | 0.00156 | up | 1.5002724 | *PYURF* |
| ENSG00000255587 | 0.00092 | up | 1.5015159 | *RAB44* |
| ENSG00000169750 | 0.00314 | up | 1.7197171 | *RAC3* |
| ENSG00000258769 | 0.03120 | up | 1.6429964 | *RAP1AP* |
| ENSG00000182732 | 0.00066 | up | 1.6918452 | *RGS6* |
| ENSG00000207005 | 0.00086 | up | 2.7023573 | *RNU1-2* |
| ENSG00000202337 | 0.04500 | up | 2.409782 | *RNU6-8* |
| ENSG00000230734 | 0.03274 | up | 2.0010574 | *RPL10P3* |
| ENSG00000134321 | 0.00173 | up | 1.5764971 | *RSAD2* |
| ENSG00000139970 | 0.00000 | up | 2.0672662 | *RTN1* |
| ENSG00000156253 | 0.00317 | up | 1.666789 | *RWDD2B* |
| ENSG00000198838 | 0.00104 | up | 1.9032531 | *RYR3* |
| ENSG00000188643 | 0.00175 | up | 1.5583618 | *S100A16* |
| ENSG00000177570 | 0.00497 | up | 2.1010504 | *SAMD12* |
| ENSG00000168077 | 0.00010 | up | 1.9617499 | *SCARA3* |
| ENSG00000252690 | 0.00051 | up | 1.5980159 | *SCARNA15* |
| ENSG00000168497 | 0.00217 | up | 1.83511 | *SDPR* |
| **ENSG00000174175** | **0.04130** | **up** | **4.1896214** | ***SELP*** |
| ENSG00000166192 | 0.01046 | up | 1.8775527 | *SENP8* |
| ENSG00000104611 | 0.00105 | up | 2.7422533 | *SH2D4A* |
| ENSG00000161681 | 0.03007 | up | 1.7212546 | *SHANK1* |
| ENSG00000163406 | 0.01142 | up | 1.5695654 | *SLC15A2* |
| ENSG00000147454 | 0.00000 | up | 2.0061743 | *SLC25A37* |
| ENSG00000129353 | 0.00025 | up | 1.6187993 | *SLC44A2* |
| ENSG00000139508 | 0.01621 | up | 1.6675838 | *SLC46A3* |
| ENSG00000004939 | 0.00000 | up | 2.3061423 | *SLC4A1* |
| ENSG00000092068 | 0.01929 | up | 1.6091868 | *SLC7A8* |
| ENSG00000137491 | 0.00000 | up | 4.9229145 | *SLCO2B1* |
| ENSG00000176463 | 0.00015 | up | 2.0986676 | *SLCO3A1* |
| ENSG00000119705 | 0.03264 | up | 1.5166293 | *SLIRP* |
| ENSG00000179542 | 0.02981 | up | 4.6416783 | *SLITRK4* |
| ENSG00000111850 | 0.00073 | up | 1.5611564 | *SMIM8* |
| ENSG00000145335 | 0.00002 | up | 1.5974464 | *SNCA* |
| ENSG00000142168 | 0.00204 | up | 1.5361028 | *SOD1* |
| ENSG00000152253 | 0.00001 | up | 1.732327 | *SPC25* |
| ENSG00000186787 | 0.00119 | up | 1.6364766 | *SPIN2B* |
| ENSG00000183018 | 0.00004 | up | 1.770038 | *SPNS2* |
| ENSG00000118785 | 0.01951 | up | 1.8597633 | *SPP1* |
| ENSG00000116096 | 0.00051 | up | 2.0500524 | *SPR* |
| ENSG00000070182 | 0.00000 | up | 1.6028488 | *SPTB* |
| ENSG00000196220 | 0.01134 | up | 3.1622913 | *SRGAP3* |
| ENSG00000234322 | 0.00683 | up | 1.6136351 | *ST13P18* |
| ENSG00000212789 | 0.00153 | up | 1.6213427 | *ST13P5* |
| ENSG00000136840 | 0.00702 | up | 1.5280039 | *ST6GALNAC4* |
| ENSG00000115415 | 0.00977 | up | 1.626963 | *STAT1* |
| ENSG00000159167 | 0.00003 | up | 1.732519 | *STC1* |
| ENSG00000169689 | 0.01015 | up | 1.5437145 | *STRA13* |
| ENSG00000165124 | 0.04049 | up | 1.7185166 | *SVEP1* |
| ENSG00000111490 | 0.03830 | up | 1.5883942 | *TBC1D30* |
| ENSG00000135111 | 0.00001 | up | 2.62361 | *TBX3* |
| ENSG00000138336 | 0.02649 | up | 2.0395226 | *TET1* |
| ENSG00000090447 | 0.00608 | up | 1.8465416 | *TFAP4* |
| ENSG00000029639 | 0.04011 | up | 1.5667119 | *TFB1M* |
| ENSG00000105825 | 0.00246 | up | 2.0978465 | *TFPI2* |
| ENSG00000119699 | 0.00088 | up | 1.9565899 | *TGFB3* |
| ENSG00000185875 | 0.01853 | up | 1.5062746 | *THNSL1* |
| ENSG00000151500 | 0.03454 | up | 1.5716397 | *THYN1* |
| ENSG00000150779 | 0.00422 | up | 1.661908 | *TIMM8B* |
| ENSG00000224908 | 0.01617 | up | 2.5228817 | *TIMM8BP2* |
| ENSG00000103534 | 0.00012 | up | 1.8623891 | *TMC5* |
| ENSG00000171204 | 0.00026 | up | 1.518672 | *TMEM126B* |
| ENSG00000168890 | 0.00028 | up | 1.5432746 | *TMEM150A* |
| ENSG00000152128 | 0.00001 | up | 1.9389439 | *TMEM163* |
| ENSG00000144120 | 0.00061 | up | 2.1781685 | *TMEM177* |
| ENSG00000185475 | 0.00337 | up | 1.6286504 | *TMEM179B* |
| ENSG00000188760 | 0.00355 | up | 1.6584263 | *TMEM198* |
| ENSG00000137038 | 0.01455 | up | 1.6293738 | *TMEM261* |
| ENSG00000135211 | 0.00021 | up | 1.6914512 | *TMEM60* |
| ENSG00000109084 | 0.00043 | up | 1.5139631 | *TMEM97* |
| ENSG00000198092 | 0.03212 | up | 2.2329688 | *TMPRSS11F* |
| ENSG00000163154 | 0.00090 | up | 1.8968084 | *TNFAIP8L2* |
| ENSG00000215788 | 0.00048 | up | 1.8486719 | *TNFRSF25* |
| ENSG00000186283 | 0.00000 | up | 1.8945392 | *TOR3A* |
| ENSG00000124496 | 0.00765 | up | 1.5893488 | *TRERF1* |
| ENSG00000121236 | 0.00137 | up | 1.812223 | *TRIM6* |
| ENSG00000154743 | 0.00032 | up | 1.6097803 | *TSEN2* |
| ENSG00000182463 | 0.00021 | up | 2.008288 | *TSHZ2* |
| ENSG00000168785 | 0.00173 | up | 1.8235681 | *TSPAN5* |
| ENSG00000180543 | 0.02937 | up | 3.4592035 | *TSPYL5* |
| ENSG00000162222 | 0.00000 | up | 1.7993387 | *TTC9C* |
| ENSG00000226147 | 0.04886 | up | 2.1077576 | *TUBBP10* |
| ENSG00000197763 | 0.02124 | up | 1.6603633 | *TXNRD3* |
| ENSG00000247240 | 0.00597 | up | 1.9538023 | *UBL7-AS1* |
| ENSG00000184076 | 0.00212 | up | 1.5765634 | *UQCR10* |
| ENSG00000126088 | 0.00325 | up | 1.5430759 | *UROD* |
| ENSG00000188690 | 0.00045 | up | 1.9879067 | *UROS* |
| ENSG00000118640 | 0.00738 | up | 1.5124816 | *VAMP8* |
| ENSG00000106018 | 0.03288 | up | 1.7172805 | *VIPR2* |
| ENSG00000103489 | 0.01145 | up | 1.7158507 | *XYLT1* |
| ENSG00000188707 | 0.00002 | up | 1.7174232 | *ZBED6CL* |
| ENSG00000102383 | 0.02094 | up | 2.4296741 | *ZDHHC15* |
| ENSG00000136367 | 0.00005 | up | 1.5060599 | *ZFHX2* |
| ENSG00000142065 | 0.04317 | up | 1.8670697 | *ZFP14* |
| ENSG00000167840 | 0.02131 | up | 1.5229228 | *ZNF232* |
| ENSG00000251666 | 0.03863 | up | 3.2298262 | *ZNF346-IT1* |
| ENSG00000197013 | 0.02139 | up | 1.5658293 | *ZNF429* |
| ENSG00000168916 | 0.00254 | up | 2.551304 | *ZNF608* |
| ENSG00000170396 | 0.00381 | up | 2.429598 | *ZNF804A* |
| ENSG00000229956 | 0.02527 | up | 3.8282409 | *ZRANB2-AS2* |
| ENSG00000184702 | 0.02379 | down | -1.6829638 | *SEPT5* |
| ENSG00000173838 | 0.00002 | down | -1.6333665 | *MARCH10* |
| ENSG00000148584 | 0.00111 | down | -2.150135 | *A1CF* |
| ENSG00000181409 | 0.00193 | down | -2.8297386 | *AATK* |
| ENSG00000165029 | 0.00724 | down | -2.6287394 | *ABCA1* |
| ENSG00000154258 | 0.00479 | down | -3.207799 | *ABCA9* |
| ENSG00000261524 | 0.01167 | down | -1.8615191 | *ABCB10P3* |
| ENSG00000260053 | 0.00379 | down | -1.8337176 | *ABCB10P4* |
| ENSG00000005471 | 0.01436 | down | -1.633363 | *ABCB4* |
| ENSG00000243064 | 0.00087 | down | -1.7001681 | *ABCC13* |
| ENSG00000023839 | 0.00089 | down | -2.1760068 | *ABCC2* |
| ENSG00000108846 | 0.00216 | down | -1.7008715 | *ABCC3* |
| ENSG00000256340 | 0.01281 | down | -2.1992497 | *ABCC6P1* |
| ENSG00000225293 | 0.03096 | down | -2.0809522 | *ABCD1P4* |
| ENSG00000160179 | 0.00009 | down | -3.3916392 | *ABCG1* |
| ENSG00000099204 | 0.00003 | down | -1.7394176 | *ABLIM1* |
| ENSG00000159842 | 0.00040 | down | -1.6101254 | *ABR* |
| ENSG00000167315 | 0.00044 | down | -1.6152138 | *ACAA2* |
| ENSG00000087085 | 0.00884 | down | -2.5319066 | *ACHE* |
| ENSG00000147174 | 0.00445 | down | -1.7888018 | *ACRC* |
| ENSG00000077522 | 0.00019 | down | -2.8718314 | *ACTN2* |
| ENSG00000135503 | 0.00469 | down | -1.5392625 | *ACVR1B* |
| ENSG00000073670 | 0.00103 | down | -1.8987113 | *ADAM11* |
| ENSG00000135074 | 0.00038 | down | -1.8708857 | *ADAM19* |
| ENSG00000140470 | 0.02406 | down | -1.646284 | *ADAMTS17* |
| ENSG00000087116 | 0.00079 | down | -3.7927456 | *ADAMTS2* |
| ENSG00000143382 | 0.00056 | down | -2.029103 | *ADAMTSL4* |
| ENSG00000147576 | 0.00013 | down | -2.73999 | *ADHFE1* |
| ENSG00000187546 | 0.00274 | down | -1.6178619 | *AGMO* |
| ENSG00000123908 | 0.00869 | down | -1.6683016 | *AGO2* |
| ENSG00000138678 | 0.00000 | down | -2.2549324 | *AGPAT9* |
| ENSG00000063438 | 0.00659 | down | -1.5001019 | *AHRR* |
| ENSG00000176092 | 0.02620 | down | -3.2243466 | *AIM1L* |
| ENSG00000163568 | 0.00794 | down | -2.1161659 | *AIM2* |
| ENSG00000131016 | 0.02233 | down | -1.8153551 | *AKAP12* |
| ENSG00000147081 | 0.00092 | down | -4.759164 | *AKAP4* |
| ENSG00000163631 | 0.00021 | down | -1.7762506 | *ALB* |
| ENSG00000118514 | 0.00109 | down | -1.6407276 | *ALDH8A1* |
| ENSG00000109107 | 0.00070 | down | -2.6123445 | *ALDOC* |
| ENSG00000171094 | 0.00094 | down | -2.1637878 | *ALK* |
| ENSG00000012779 | 0.00157 | down | -3.298984 | *ALOX5* |
| ENSG00000242110 | 0.00002 | down | -1.6136559 | *AMACR* |
| ENSG00000116748 | 0.00952 | down | -5.3907857 | *AMPD1* |
| ENSG00000174945 | 0.00004 | down | -2.2923715 | *AMZ1* |
| ENSG00000145362 | 0.00075 | down | -1.9183698 | *ANK2* |
| ENSG00000132623 | 0.00014 | down | -1.9334657 | *ANKEF1* |
| ENSG00000153930 | 0.00001 | down | -1.8347275 | *ANKFN1* |
| ENSG00000167522 | 0.00311 | down | -1.5042648 | *ANKRD11* |
| ENSG00000242676 | 0.00117 | down | -2.2090304 | *ANKRD20A12P* |
| ENSG00000154065 | 0.00008 | down | -2.168887 | *ANKRD29* |
| ENSG00000235711 | 0.01290 | down | -3.4055529 | *ANKRD34C* |
| ENSG00000259461 | 0.01057 | down | -2.9839275 | *ANP32BP3* |
| ENSG00000131480 | 0.00001 | down | -1.817179 | *AOC2* |
| ENSG00000131471 | 0.00025 | down | -2.0474002 | *AOC3* |
| ENSG00000260105 | 0.01459 | down | -2.4229817 | *AOC4P* |
| ENSG00000166313 | 0.01051 | down | -1.6552812 | *APBB1* |
| ENSG00000163697 | 0.00004 | down | -2.0826735 | *APBB2* |
| ENSG00000115266 | 0.00032 | down | -1.9103991 | *APC2* |
| ENSG00000234906 | 0.01422 | down | -1.8493705 | *APOC2* |
| ENSG00000128284 | 0.00196 | down | -1.8606362 | *APOL3* |
| ENSG00000165272 | 0.00040 | down | -1.8525004 | *AQP3* |
| ENSG00000165269 | 0.00150 | down | -2.9612741 | *AQP7* |
| ENSG00000186466 | 0.04905 | down | -3.2842393 | *AQP7P1* |
| ENSG00000156750 | 0.00118 | down | -1.9652632 | *AQP7P3* |
| ENSG00000103375 | 0.00668 | down | -2.85827 | *AQP8* |
| ENSG00000198576 | 0.00271 | down | -2.4338024 | *ARC* |
| ENSG00000109321 | 0.00043 | down | -2.0437725 | *AREG* |
| ENSG00000205595 | 0.00002 | down | -1.9988024 | *AREGB* |
| ENSG00000225485 | 0.00119 | down | -2.9891543 | *ARHGAP23* |
| ENSG00000159314 | 0.00018 | down | -1.7107208 | *ARHGAP27* |
| ENSG00000137962 | 0.01918 | down | -4.093739 | *ARHGAP29* |
| ENSG00000165895 | 0.00065 | down | -1.5897156 | *ARHGAP42* |
| ENSG00000074964 | 0.00011 | down | -2.8303826 | *ARHGEF10L* |
| ENSG00000204959 | 0.03749 | down | -1.7524898 | *ARHGEF34P* |
| ENSG00000050327 | 0.01957 | down | -1.7209026 | *ARHGEF5* |
| ENSG00000116017 | 0.00009 | down | -1.8686585 | *ARID3A* |
| ENSG00000188042 | 0.00001 | down | -2.294126 | *ARL4C* |
| ENSG00000122872 | 0.02627 | down | -2.9997196 | *ARL4P* |
| ENSG00000250151 | 0.03647 | down | -1.9032277 | *ARPC4-TTLL3* |
| ENSG00000141505 | 0.01016 | down | -2.629528 | *ASGR1* |
| ENSG00000198363 | 0.00419 | down | -1.635415 | *ASPH* |
| ENSG00000188886 | 0.02443 | down | -2.515009 | *ASTL* |
| ENSG00000162772 | 0.00001 | down | -2.1328323 | *ATF3* |
| ENSG00000213338 | 0.02698 | down | -2.1687825 | *ATF4P1* |
| ENSG00000231342 | 0.02698 | down | -2.1687825 | *ATF4P2* |
| ENSG00000256167 | 0.00157 | down | -1.5231361 | *ATF4P4* |
| ENSG00000143153 | 0.00788 | down | -1.6103493 | *ATP1B1* |
| ENSG00000196296 | 0.00011 | down | -1.9530898 | *ATP2A1* |
| ENSG00000205464 | 0.00064 | down | -1.8897904 | *ATP6AP1L* |
| ENSG00000130270 | 0.03159 | down | -2.2964625 | *ATP8B3* |
| ENSG00000124788 | 0.00171 | down | -1.6422302 | *ATXN1* |
| ENSG00000135407 | 0.00534 | down | -2.062848 | *AVIL* |
| ENSG00000160862 | 0.00660 | down | -2.0241315 | *AZGP1* |
| ENSG00000214313 | 0.00180 | down | -2.2892632 | *AZGP1P1* |
| ENSG00000198488 | 0.01776 | down | -3.9533968 | *B3GNT6* |
| ENSG00000177191 | 0.00617 | down | -3.3764284 | *B3GNT8* |
| ENSG00000182272 | 0.01281 | down | -2.3381784 | *B4GALNT4* |
| ENSG00000086062 | 0.00136 | down | -1.5068966 | *B4GALT1* |
| ENSG00000136881 | 0.02678 | down | -1.6619526 | *BAAT* |
| ENSG00000112182 | 0.00017 | down | -3.4848495 | *BACH2* |
| ENSG00000006453 | 0.01651 | down | -3.7167904 | *BAIAP2L1* |
| ENSG00000007516 | 0.00377 | down | -1.9238118 | *BAIAP3* |
| ENSG00000095739 | 0.01209 | down | -1.8492656 | *BAMBI* |
| ENSG00000105327 | 0.00257 | down | -2.2872276 | *BBC3* |
| ENSG00000262117 | 0.00283 | down | -1.8182214 | *BCAR4* |
| ENSG00000171791 | 0.00016 | down | -2.2344244 | *BCL2* |
| ENSG00000069399 | 0.00000 | down | -1.6202862 | *BCL3* |
| ENSG00000113916 | 0.00029 | down | -2.8899264 | *BCL6* |
| ENSG00000186174 | 0.01398 | down | -1.5047159 | *BCL9L* |
| ENSG00000183337 | 0.01019 | down | -1.598706 | *BCOR* |
| ENSG00000166546 | 0.00202 | down | -6.3111615 | *BEAN1* |
| ENSG00000134107 | 0.00045 | down | -1.6598839 | *BHLHE40* |
| ENSG00000136717 | 0.00623 | down | -1.9992896 | *BIN1* |
| ENSG00000104081 | 0.00002 | down | -1.8363923 | *BMF* |
| ENSG00000125378 | 0.00000 | down | -2.8442523 | *BMP4* |
| ENSG00000152430 | 0.03897 | down | -4.3773265 | *BOLL* |
| ENSG00000222009 | 0.00003 | down | -1.5140464 | *BTBD19* |
| ENSG00000185522 | 0.01598 | down | -1.6104592 | *C11orf35* |
| ENSG00000188596 | 0.00754 | down | -3.5690296 | *C12orf55* |
| ENSG00000257242 | 0.01491 | down | -2.4825644 | *C12orf79* |
| ENSG00000188277 | 0.02391 | down | -1.8818226 | *C15orf62* |
| ENSG00000182831 | 0.00208 | down | -1.5908248 | *C16orf72* |
| ENSG00000205832 | 0.01430 | down | -2.7099485 | *C16orf96* |
| ENSG00000261359 | 0.03267 | down | -2.7118626 | *C16orf98* |
| ENSG00000154035 | 0.02063 | down | -1.6285975 | *C17orf103* |
| ENSG00000214212 | 0.02824 | down | -1.5200425 | *C19orf38* |
| ENSG00000183397 | 0.02002 | down | -1.9078567 | *C19orf71* |
| ENSG00000095932 | 0.00658 | down | -4.7924075 | *C19orf77* |
| ENSG00000163263 | 0.01931 | down | -2.5043232 | *C1orf189* |
| ENSG00000182326 | 0.01344 | down | -1.9938743 | *C1S* |
| ENSG00000166278 | 0.00749 | down | -2.2262516 | *C2* |
| ENSG00000163009 | 0.01118 | down | -1.5868137 | *C2orf48* |
| ENSG00000042304 | 0.03244 | down | -4.871109 | *C2orf83* |
| ENSG00000197405 | 0.00030 | down | -3.0784397 | *C5AR1* |
| ENSG00000188112 | 0.01747 | down | -2.061562 | *C6orf132* |
| ENSG00000189325 | 0.00001 | down | -3.8696482 | *C6orf222* |
| ENSG00000176907 | 0.01955 | down | -2.8247428 | *C8orf4* |
| ENSG00000232434 | 0.00593 | down | -2.1319292 | *C9orf172* |
| ENSG00000197768 | 0.00095 | down | -3.6971843 | *C9orf173* |
| ENSG00000164879 | 0.01486 | down | -1.590012 | *CA3* |
| ENSG00000107159 | 0.00325 | down | -3.090994 | *CA9* |
| ENSG00000154040 | 0.00637 | down | -2.6676168 | *CABYR* |
| ENSG00000007402 | 0.02806 | down | -2.1066277 | *CACNA2D2* |
| ENSG00000130433 | 0.01127 | down | -2.945292 | *CACNG6* |
| ENSG00000004660 | 0.00012 | down | -1.8759286 | *CAMKK1* |
| ENSG00000076826 | 0.00046 | down | -3.0506387 | *CAMSAP3* |
| ENSG00000198286 | 0.00104 | down | -4.4810762 | *CARD11* |
| ENSG00000166762 | 0.03499 | down | -1.5108339 | *CATSPER2* |
| ENSG00000141668 | 0.00065 | down | -2.2626886 | *CBLN2* |
| ENSG00000172361 | 0.00492 | down | -2.1050165 | *CCDC11* |
| ENSG00000161180 | 0.03470 | down | -3.2402596 | *CCDC116* |
| ENSG00000255181 | 0.00006 | down | -5.9014606 | *CCDC166* |
| ENSG00000130783 | 0.00083 | down | -1.7494746 | *CCDC62* |
| ENSG00000253276 | 0.00655 | down | -1.594914 | *CCDC71L* |
| ENSG00000169515 | 0.01348 | down | -3.152665 | *CCDC8* |
| ENSG00000055813 | 0.00000 | down | -2.7097745 | *CCDC85A* |
| ENSG00000138764 | 0.00717 | down | -1.6849163 | *CCNG2* |
| ENSG00000121807 | 0.00456 | down | -3.5130796 | *CCR2* |
| ENSG00000170458 | 0.00331 | down | -1.8868041 | *CD14* |
| ENSG00000120217 | 0.00463 | down | -2.585355 | *CD274* |
| ENSG00000149798 | 0.00050 | down | -1.5908595 | *CDC42EP2* |
| ENSG00000081138 | 0.00072 | down | -1.92848 | *CDH7* |
| ENSG00000176749 | 0.00111 | down | -1.7309949 | *CDK5R1* |
| ENSG00000171450 | 0.00005 | down | -3.4559238 | *CDK5R2* |
| ENSG00000129757 | 0.00003 | down | -1.8237402 | *CDKN1C* |
| ENSG00000079385 | 0.00680 | down | -2.2841718 | *CEACAM1* |
| ENSG00000172216 | 0.02448 | down | -1.5426133 | *CEBPB* |
| ENSG00000099954 | 0.00090 | down | -4.042623 | *CECR2* |
| ENSG00000159409 | 0.02008 | down | -2.1917095 | *CELF3* |
| ENSG00000161082 | 0.00864 | down | -4.2825537 | *CELF5* |
| ENSG00000099814 | 0.00182 | down | -2.076695 | *CEP170B* |
| ENSG00000154227 | 0.00093 | down | -2.7434657 | *CERS3* |
| ENSG00000087237 | 0.00010 | down | -1.8130635 | *CETP* |
| ENSG00000126759 | 0.03791 | down | -1.5575444 | *CFP* |
| ENSG00000100399 | 0.00770 | down | -1.8691508 | *CHADL* |
| ENSG00000166664 | 0.01882 | down | -1.6136916 | *CHRFAM7A* |
| ENSG00000175344 | 0.04331 | down | -1.6599193 | *CHRNA7* |
| ENSG00000154080 | 0.03031 | down | -2.060637 | *CHST9* |
| ENSG00000230304 | 0.03468 | down | -2.4862778 | *CICP6* |
| ENSG00000160161 | 0.00046 | down | -2.4195223 | *CILP2* |
| ENSG00000016490 | 0.00000 | down | -1.8466705 | *CLCA1* |
| ENSG00000137975 | 0.00002 | down | -2.489822 | *CLCA2* |
| ENSG00000213937 | 0.03083 | down | -3.1320987 | *CLDN9* |
| ENSG00000157322 | 0.00426 | down | -1.6749151 | *CLEC18A* |
| ENSG00000140839 | 0.00001 | down | -1.9639626 | *CLEC18B* |
| ENSG00000157335 | 0.00731 | down | -1.5123947 | *CLEC18C* |
| ENSG00000115295 | 0.00756 | down | -1.9039627 | *CLIP4* |
| ENSG00000079101 | 0.00699 | down | -4.9223585 | *CLUL1* |
| ENSG00000184144 | 0.00177 | down | -1.8187987 | *CNTN2* |
| ENSG00000154529 | 0.00022 | down | -2.3946302 | *CNTNAP3B* |
| ENSG00000204248 | 0.00006 | down | -1.9505894 | *COL11A2* |
| ENSG00000101203 | 0.02189 | down | -7.2310286 | *COL20A1* |
| ENSG00000188153 | 0.00163 | down | -1.7617325 | *COL4A5* |
| ENSG00000204262 | 0.00251 | down | -2.0500784 | *COL5A2* |
| ENSG00000163359 | 0.03635 | down | -2.0354493 | *COL6A3* |
| ENSG00000114270 | 0.00481 | down | -1.6969608 | *COL7A1* |
| ENSG00000171812 | 0.02057 | down | -3.4709928 | *COL8A2* |
| ENSG00000092758 | 0.00141 | down | -3.2495093 | *COL9A3* |
| ENSG00000145244 | 0.00075 | down | -2.1177676 | *CORIN* |
| ENSG00000160471 | 0.00023 | down | -2.1665664 | *COX6B2* |
| ENSG00000168993 | 0.01622 | down | -2.0885148 | *CPLX1* |
| ENSG00000213578 | 0.00063 | down | -3.1879797 | *CPLX3* |
| ENSG00000109625 | 0.01000 | down | -1.9659616 | *CPZ* |
| ENSG00000117322 | 0.00047 | down | -1.9500304 | *CR2* |
| ENSG00000146592 | 0.00753 | down | -1.6384174 | *CREB5* |
| ENSG00000164463 | 0.00178 | down | -2.074087 | *CREBRF* |
| ENSG00000095794 | 0.00004 | down | -1.715837 | *CREM* |
| ENSG00000213145 | 0.01453 | down | -4.0273914 | *CRIP1* |
| ENSG00000182809 | 0.00487 | down | -1.9105287 | *CRIP2* |
| ENSG00000103196 | 0.04355 | down | -1.7290611 | *CRISPLD2* |
| ENSG00000184324 | 0.00112 | down | -7.18078 | *CSAG2* |
| ENSG00000197463 | 0.00100 | down | -7.312831 | *CSAG3* |
| ENSG00000172346 | 0.04306 | down | -2.5427985 | *CSDC2* |
| ENSG00000183117 | 0.01411 | down | -2.457231 | *CSMD1* |
| ENSG00000114646 | 0.03853 | down | -2.1376739 | *CSPG5* |
| ENSG00000144655 | 0.00031 | down | -2.34461 | *CSRNP1* |
| ENSG00000226907 | 0.00012 | down | -1.547845 | *CT45A6* |
| ENSG00000169862 | 0.00340 | down | -2.568772 | *CTNND2* |
| ENSG00000140465 | 0.01231 | down | -1.6320692 | *CYP1A1* |
| ENSG00000204338 | 0.00997 | down | -3.4555776 | *CYP21A1P* |
| ENSG00000165841 | 0.00416 | down | -2.758365 | *CYP2C19* |
| ENSG00000197446 | 0.04153 | down | -2.6042514 | *CYP2F1* |
| ENSG00000167600 | 0.00029 | down | -2.374816 | *CYP2S1* |
| ENSG00000108669 | 0.00064 | down | -1.6280683 | *CYTH1* |
| ENSG00000100055 | 0.00008 | down | -1.5790305 | *CYTH4* |
| ENSG00000115165 | 0.00001 | down | -1.7424419 | *CYTIP* |
| ENSG00000035664 | 0.00000 | down | -2.2159548 | *DAPK2* |
| ENSG00000182308 | 0.00208 | down | -1.6249261 | *DCAF4L1* |
| ENSG00000226372 | 0.00000 | down | -2.9725332 | *DCAF8L1* |
| ENSG00000198924 | 0.00004 | down | -1.6732314 | *DCLRE1A* |
| ENSG00000163354 | 0.00002 | down | -2.292294 | *DCST2* |
| ENSG00000145358 | 0.02762 | down | -3.3877225 | *DDIT4L* |
| ENSG00000181418 | 0.01322 | down | -2.127048 | *DDN* |
| ENSG00000204580 | 0.00055 | down | -2.1471841 | *DDR1* |
| ENSG00000227159 | 0.04166 | down | -2.2322092 | *DDX11L16* |
| ENSG00000175084 | 0.01845 | down | -5.8562293 | *DES* |
| ENSG00000205267 | 0.02400 | down | -4.4035983 | *DGAT2L7P* |
| ENSG00000104808 | 0.01433 | down | -2.6837997 | *DHDH* |
| ENSG00000162496 | 0.00889 | down | -1.8551944 | *DHRS3* |
| ENSG00000115844 | 0.02982 | down | -3.33516 | *DLX2* |
| ENSG00000198947 | 0.00159 | down | -1.9660672 | *DMD* |
| ENSG00000104936 | 0.00621 | down | -1.6109539 | *DMPK* |
| ENSG00000142025 | 0.00722 | down | -2.9782677 | *DMRTC2* |
| ENSG00000185800 | 0.00238 | down | -1.6588974 | *DMWD* |
| ENSG00000114841 | 0.01943 | down | -1.6630341 | *DNAH1* |
| ENSG00000187775 | 0.00169 | down | -1.7048887 | *DNAH17* |
| ENSG00000124721 | 0.00046 | down | -2.0466013 | *DNAH8* |
| ENSG00000128590 | 0.00019 | down | -1.5317525 | *DNAJB9* |
| ENSG00000178401 | 0.00479 | down | -1.8592044 | *DNAJC22* |
| ENSG00000235370 | 0.04086 | down | -2.094151 | *DNM1P51* |
| ENSG00000107554 | 0.00009 | down | -2.432976 | *DNMBP* |
| ENSG00000088538 | 0.00236 | down | -1.7289352 | *DOCK3* |
| ENSG00000104885 | 0.00154 | down | -1.6115155 | *DOT1L* |
| ENSG00000188641 | 0.00393 | down | -2.1893585 | *DPYD* |
| ENSG00000157856 | 0.01639 | down | -3.5511298 | *DRC1* |
| ENSG00000096696 | 0.00935 | down | -3.3435643 | *DSP* |
| ENSG00000178498 | 0.00388 | down | -1.5588264 | *DTX3* |
| ENSG00000120129 | 0.00002 | down | -1.8610256 | *DUSP1* |
| ENSG00000143507 | 0.00000 | down | -1.8563573 | *DUSP10* |
| ENSG00000111266 | 0.00040 | down | -2.9422393 | *DUSP16* |
| ENSG00000138166 | 0.00025 | down | -1.7798805 | *DUSP5* |
| ENSG00000139318 | 0.00000 | down | -1.9542419 | *DUSP6* |
| ENSG00000184545 | 0.00274 | down | -3.5494611 | *DUSP8* |
| ENSG00000259029 | 0.00163 | down | -2.654602 | *DUX4L18* |
| ENSG00000224807 | 0.02540 | down | -3.8295953 | *DUX4L9* |
| ENSG00000165891 | 0.00162 | down | -1.6309582 | *E2F7* |
| ENSG00000101210 | 0.00684 | down | -2.5161567 | *EEF1A2* |
| ENSG00000115468 | 0.00211 | down | -1.9801105 | *EFHD1* |
| ENSG00000125266 | 0.00251 | down | -1.6375072 | *EFNB2* |
| ENSG00000135766 | 0.00103 | down | -1.5163298 | *EGLN1* |
| ENSG00000120738 | 0.00001 | down | -3.1995573 | *EGR1* |
| ENSG00000122877 | 0.00016 | down | -3.0017931 | *EGR2* |
| ENSG00000179388 | 0.00000 | down | -2.2614615 | *EGR3* |
| ENSG00000173442 | 0.00316 | down | -1.7079929 | *EHBP1L1* |
| ENSG00000163435 | 0.00938 | down | -3.6182084 | *ELF3* |
| ENSG00000225968 | 0.00447 | down | -2.5958064 | *ELFN1* |
| ENSG00000166897 | 0.01121 | down | -1.6135848 | *ELFN2* |
| ENSG00000164035 | 0.02747 | down | -2.742214 | *EMCN* |
| ENSG00000134531 | 0.00000 | down | -2.5219836 | *EMP1* |
| ENSG00000213853 | 0.00048 | down | -2.4235091 | *EMP2* |
| ENSG00000111674 | 0.00411 | down | -1.7514101 | *ENO2* |
| ENSG00000164303 | 0.00000 | down | -1.801363 | *ENPP6* |
| ENSG00000106123 | 0.00010 | down | -2.5230324 | *EPHB6* |
| ENSG00000227184 | 0.00013 | down | -3.878863 | *EPPK1* |
| ENSG00000131037 | 0.02085 | down | -1.6430707 | *EPS8L1* |
| ENSG00000177106 | 0.01235 | down | -2.045823 | *EPS8L2* |
| ENSG00000157554 | 0.00022 | down | -1.5818634 | *ERG* |
| ENSG00000136541 | 0.00027 | down | -1.8648422 | *ERMN* |
| ENSG00000178607 | 0.00379 | down | -1.8069389 | *ERN1* |
| ENSG00000116285 | 0.00055 | down | -1.7254664 | *ERRFI1* |
| ENSG00000144488 | 0.00223 | down | -3.0584817 | *ESPNL* |
| ENSG00000268869 | 0.00173 | down | -2.5388117 | *ESPNP* |
| ENSG00000134954 | 0.02470 | down | -1.8465936 | *ETS1* |
| ENSG00000244405 | 0.01071 | down | -1.5028915 | *ETV5* |
| ENSG00000072840 | 0.00501 | down | -1.8761584 | *EVC* |
| ENSG00000173040 | 0.00428 | down | -2.2129612 | *EVC2* |
| ENSG00000185862 | 0.01048 | down | -1.7798543 | *EVI2B* |
| ENSG00000092820 | 0.00000 | down | -1.6481735 | *EZR* |
| ENSG00000183688 | 0.00002 | down | -2.0775914 | *FAM101B* |
| ENSG00000198420 | 0.00001 | down | -1.9555554 | *FAM115A* |
| ENSG00000170379 | 0.00184 | down | -1.7186332 | *FAM115C* |
| ENSG00000159860 | 0.00368 | down | -1.6846073 | *FAM115D* |
| ENSG00000175182 | 0.03719 | down | -2.108393 | *FAM131A* |
| ENSG00000138640 | 0.00014 | down | -1.9838346 | *FAM13A* |
| ENSG00000248019 | 0.00000 | down | -2.300536 | *FAM13A-AS1* |
| ENSG00000161682 | 0.00833 | down | -1.8988155 | *FAM171A2* |
| ENSG00000168754 | 0.01803 | down | -2.5083156 | *FAM178B* |
| ENSG00000189320 | 0.00486 | down | -2.3082497 | *FAM180A* |
| ENSG00000214447 | 0.02303 | down | -1.9041489 | *FAM187A* |
| ENSG00000135063 | 0.00096 | down | -1.8569493 | *FAM189A2* |
| ENSG00000112773 | 0.00089 | down | -1.5663503 | *FAM46A* |
| ENSG00000187773 | 0.00010 | down | -2.3962038 | *FAM69C* |
| ENSG00000162771 | 0.00711 | down | -2.3218083 | *FAM71A* |
| ENSG00000125998 | 0.00757 | down | -3.3365142 | *FAM83C* |
| ENSG00000105523 | 0.02317 | down | -2.1585681 | *FAM83E* |
| ENSG00000133477 | 0.00001 | down | -2.3442311 | *FAM83F* |
| ENSG00000188522 | 0.00600 | down | -1.5052099 | *FAM83G* |
| ENSG00000180921 | 0.00217 | down | -1.7133409 | *FAM83H* |
| ENSG00000203499 | 0.02210 | down | -1.5556628 | *FAM83H-AS1* |
| ENSG00000162981 | 0.00040 | down | -2.3152082 | *FAM84A* |
| ENSG00000168672 | 0.00230 | down | -1.7187928 | *FAM84B* |
| ENSG00000230342 | 0.00855 | down | -1.8306413 | *FANCD2P2* |
| ENSG00000117560 | 0.00412 | down | -2.093277 | *FASLG* |
| ENSG00000156804 | 0.00709 | down | -2.2626274 | *FBXO32* |
| ENSG00000109670 | 0.00199 | down | -1.6377294 | *FBXW7* |
| ENSG00000130475 | 0.01284 | down | -2.207171 | *FCHO1* |
| ENSG00000132185 | 0.01781 | down | -4.0132213 | *FCRLA* |
| ENSG00000162746 | 0.01942 | down | -1.7845486 | *FCRLB* |
| ENSG00000088340 | 0.00133 | down | -3.4032981 | *FER1L4* |
| ENSG00000146192 | 0.03302 | down | -2.8086135 | *FGD2* |
| ENSG00000156427 | 0.00218 | down | -2.3109787 | *FGF18* |
| ENSG00000160867 | 0.01113 | down | -1.582353 | *FGFR4* |
| ENSG00000171557 | 0.02534 | down | -2.2396853 | *FGG* |
| ENSG00000137460 | 0.01146 | down | -1.5763416 | *FHDC1* |
| ENSG00000125848 | 0.03030 | down | -5.153321 | *FLRT3* |
| ENSG00000102755 | 0.03780 | down | -2.898323 | *FLT1* |
| ENSG00000090554 | 0.00513 | down | -1.6052736 | *FLT3LG* |
| ENSG00000184922 | 0.00138 | down | -1.6848948 | *FMNL1* |
| ENSG00000170345 | 0.00003 | down | -2.557217 | *FOS* |
| ENSG00000125740 | 0.00000 | down | -4.4836154 | *FOSB* |
| ENSG00000129514 | 0.00309 | down | -2.6448 | *FOXA1* |
| ENSG00000187140 | 0.00031 | down | -2.89518 | *FOXD3* |
| ENSG00000150907 | 0.01111 | down | -2.004352 | *FOXO1* |
| ENSG00000150893 | 0.01938 | down | -3.4137566 | *FREM2* |
| ENSG00000205097 | 0.01496 | down | -3.650721 | *FRG2* |
| ENSG00000172969 | 0.00008 | down | -4.234592 | *FRG2C* |
| ENSG00000075618 | 0.00003 | down | -1.7824858 | *FSCN1* |
| ENSG00000150201 | 0.02684 | down | -2.3891768 | *FXYD4* |
| ENSG00000139112 | 0.00005 | down | -1.7796527 | *GABARAPL1* |
| ENSG00000166206 | 0.00113 | down | -2.215167 | *GABRB3* |
| ENSG00000099860 | 0.03684 | down | -1.6238427 | *GADD45B* |
| ENSG00000154252 | 0.04086 | down | -2.7998302 | *GAL3ST2* |
| ENSG00000249210 | 0.00362 | down | -2.8181014 | *GAPDHP38* |
| ENSG00000248626 | 0.01461 | down | -2.586335 | *GAPDHP40* |
| ENSG00000219881 | 0.00000 | down | -2.372618 | *GAPDHP42* |
| ENSG00000141441 | 0.01004 | down | -2.1425896 | *GAREM* |
| ENSG00000180447 | 0.02654 | down | -2.6181848 | *GAS1* |
| ENSG00000141013 | 0.00807 | down | -1.5106108 | *GAS8* |
| ENSG00000141448 | 0.03732 | down | -5.6490874 | *GATA6* |
| ENSG00000143614 | 0.00054 | down | -1.792072 | *GATAD2B* |
| ENSG00000164900 | 0.00269 | down | -2.0898643 | *GBX1* |
| ENSG00000130513 | 0.00135 | down | -2.3730843 | *GDF15* |
| ENSG00000164949 | 0.00239 | down | -2.8437364 | *GEM* |
| ENSG00000131459 | 0.00000 | down | -1.5801967 | *GFPT2* |
| ENSG00000100031 | 0.00595 | down | -1.6955328 | *GGT1* |
| ENSG00000100121 | 0.01515 | down | -2.4149542 | *GGTLC2* |
| ENSG00000121853 | 0.00010 | down | -2.9598618 | *GHSR* |
| ENSG00000165474 | 0.00121 | down | -1.7758249 | *GJB2* |
| ENSG00000182963 | 0.00273 | down | -2.181089 | *GJC1* |
| ENSG00000111087 | 0.00461 | down | -2.8519273 | *GLI1* |
| ENSG00000107249 | 0.00139 | down | -2.163381 | *GLIS3* |
| ENSG00000112164 | 0.02862 | down | -2.6758034 | *GLP1R* |
| ENSG00000250959 | 0.00235 | down | -1.9209843 | *GLUD1P3* |
| ENSG00000069966 | 0.00085 | down | -1.5237246 | *GNB5* |
| ENSG00000125787 | 0.03499 | down | -2.3220105 | *GNRH2* |
| ENSG00000090615 | 0.00026 | down | -1.5480504 | *GOLGA3* |
| ENSG00000215186 | 0.01671 | down | -3.6901996 | *GOLGA6B* |
| ENSG00000215252 | 0.00936 | down | -1.5783454 | *GOLGA8B* |
| ENSG00000181984 | 0.00303 | down | -2.1934636 | *GOLGA8CP* |
| ENSG00000153684 | 0.01117 | down | -2.882532 | *GOLGA8F* |
| ENSG00000183629 | 0.00715 | down | -2.7883682 | *GOLGA8G* |
| ENSG00000174567 | 0.02016 | down | -2.7058692 | *GOLT1A* |
| ENSG00000178732 | 0.02020 | down | -2.7029893 | *GP5* |
| ENSG00000063660 | 0.02155 | down | -1.7102243 | *GPC1* |
| ENSG00000235984 | 0.00489 | down | -5.1142197 | *GPC5-AS1* |
| ENSG00000183098 | 0.00017 | down | -2.2516575 | *GPC6* |
| ENSG00000112414 | 0.00005 | down | -2.0012434 | *GPR126* |
| ENSG00000155269 | 0.01473 | down | -1.8802168 | *GPR78* |
| ENSG00000013588 | 0.00015 | down | -2.3470054 | *GPRC5A* |
| ENSG00000167191 | 0.00346 | down | -4.9237595 | *GPRC5B* |
| ENSG00000161835 | 0.04743 | down | -2.2879834 | *GRASP* |
| ENSG00000134317 | 0.00197 | down | -1.7645469 | *GRHL1* |
| ENSG00000155974 | 0.00136 | down | -1.7101042 | *GRIP1* |
| ENSG00000189369 | 0.00083 | down | -1.7719342 | *GSPT2* |
| ENSG00000065621 | 0.00600 | down | -2.1070716 | *GSTO2* |
| ENSG00000242441 | 0.00017 | down | -2.038914 | *GTF2A1L* |
| ENSG00000170627 | 0.01594 | down | -1.6370146 | *GTSF1* |
| ENSG00000101890 | 0.00273 | down | -2.3413897 | *GUCY2F* |
| ENSG00000056998 | 0.00103 | down | -3.9613156 | *GYG2* |
| ENSG00000162882 | 0.02104 | down | -3.2992837 | *HAAO* |
| ENSG00000132702 | 0.00252 | down | -3.6425602 | *HAPLN2* |
| ENSG00000140511 | 0.00470 | down | -2.9705431 | *HAPLN3* |
| ENSG00000113070 | 0.00001 | down | -2.6160378 | *HBEGF* |
| ENSG00000103145 | 0.00851 | down | -1.724211 | *HCFC1R1* |
| ENSG00000101336 | 0.00164 | down | -4.616454 | *HCK* |
| ENSG00000099822 | 0.01845 | down | -2.043532 | *HCN2* |
| ENSG00000048052 | 0.00027 | down | -2.6805398 | *HDAC9* |
| ENSG00000112273 | 0.00208 | down | -3.727292 | *HDGFL1* |
| ENSG00000112406 | 0.00171 | down | -2.10657 | *HECA* |
| ENSG00000173706 | 0.00011 | down | -2.0074687 | *HEG1* |
| ENSG00000188175 | 0.00008 | down | -2.566956 | *HEPACAM2* |
| ENSG00000230267 | 0.00303 | down | -1.9126784 | *HERC2P4* |
| ENSG00000260644 | 0.00324 | down | -1.8940284 | *HERC2P5* |
| ENSG00000261599 | 0.01010 | down | -1.9775212 | *HERC2P8* |
| ENSG00000113924 | 0.00056 | down | -1.8642911 | *HGD* |
| ENSG00000177374 | 0.00059 | down | -1.7756467 | *HIC1* |
| ENSG00000160396 | 0.01544 | down | -4.122824 | *HIPK4* |
| ENSG00000168298 | 0.04602 | down | -2.215002 | *HIST1H1E* |
| ENSG00000196866 | 0.01916 | down | -1.5693412 | *HIST1H2AD* |
| ENSG00000198374 | 0.02627 | down | -4.05957 | *HIST1H2AL* |
| ENSG00000256018 | 0.02930 | down | -2.521937 | *HIST1H3G* |
| ENSG00000188987 | 0.03804 | down | -3.6376598 | *HIST1H4D* |
| ENSG00000198339 | 0.00695 | down | -2.722514 | *HIST1H4I* |
| ENSG00000203819 | 0.02093 | down | -1.9753584 | *HIST2H2BC* |
| ENSG00000220323 | 0.04870 | down | -2.0629115 | *HIST2H2BD* |
| ENSG00000203852 | 0.03398 | down | -2.1224194 | *HIST2H3A* |
| ENSG00000203811 | 0.03398 | down | -2.1224196 | *HIST2H3C* |
| ENSG00000183941 | 0.00283 | down | -1.6563065 | *HIST2H4A* |
| ENSG00000182217 | 0.00193 | down | -1.6752796 | *HIST2H4B* |
| ENSG00000232629 | 0.01085 | down | -2.3517058 | *HLA-DQB2* |
| ENSG00000270362 | 0.04924 | down | -2.50682 | *HMGN3-AS1* |
| ENSG00000180448 | 0.00064 | down | -1.7092918 | *HMHA1* |
| ENSG00000100292 | 0.00015 | down | -1.6548011 | *HMOX1* |
| ENSG00000231942 | 0.01527 | down | -2.5418456 | *HNRNPA1P36* |
| ENSG00000263179 | 0.02689 | down | -1.7393472 | *HNRNPCP4* |
| ENSG00000120075 | 0.02670 | down | -2.5838156 | *HOXB5* |
| ENSG00000198353 | 0.01035 | down | -2.1188698 | *HOXC4* |
| ENSG00000257017 | 0.01294 | down | -3.2777102 | *HP* |
| ENSG00000116983 | 0.00232 | down | -2.89869 | *HPCAL4* |
| ENSG00000166104 | 0.00333 | down | -1.6660634 | *hsa-mir-7162* |
| ENSG00000198857 | 0.00973 | down | -3.010189 | *HSD3BP5* |
| ENSG00000147246 | 0.00002 | down | -4.2437544 | *HTR2C* |
| ENSG00000148680 | 0.00291 | down | -2.5830867 | *HTR7* |
| ENSG00000117318 | 0.00001 | down | -2.0726595 | *ID3* |
| ENSG00000148377 | 0.00121 | down | -2.7333503 | *IDI2* |
| ENSG00000160888 | 0.01010 | down | -1.7447959 | *IER2* |
| ENSG00000137331 | 0.00046 | down | -2.3465562 | *IER3* |
| ENSG00000162783 | 0.00155 | down | -1.940159 | *IER5* |
| ENSG00000103742 | 0.00361 | down | -2.289433 | *IGDCC4* |
| ENSG00000140443 | 0.00011 | down | -2.0259597 | *IGF1R* |
| ENSG00000167244 | 0.01588 | down | -3.026078 | *IGF2* |
| ENSG00000073792 | 0.00021 | down | -2.1663015 | *IGF2BP2* |
| ENSG00000099769 | 0.02095 | down | -3.1282663 | *IGFALS* |
| ENSG00000146674 | 0.01765 | down | -3.872478 | *IGFBP3* |
| ENSG00000253895 | 0.02492 | down | -2.0900254 | *IGHVII-44-2* |
| ENSG00000085552 | 0.02208 | down | -1.852587 | *IGSF9* |
| ENSG00000123411 | 0.00032 | down | -1.8000224 | *IKZF4* |
| ENSG00000110324 | 0.00001 | down | -2.0633152 | *IL10RA* |
| ENSG00000169194 | 0.00893 | down | -4.367422 | *IL13* |
| ENSG00000115594 | 0.00282 | down | -1.6049366 | *IL1R1* |
| ENSG00000115590 | 0.04640 | down | -3.0285802 | *IL1R2* |
| ENSG00000115602 | 0.00079 | down | -2.1836634 | *IL1RL1* |
| ENSG00000136689 | 0.02908 | down | -2.114572 | *IL1RN* |
| ENSG00000103522 | 0.00003 | down | -1.8893383 | *IL21R* |
| ENSG00000259954 | 0.00021 | down | -1.9432704 | *IL21R-AS1* |
| ENSG00000134460 | 0.00001 | down | -1.6516867 | *IL2RA* |
| ENSG00000100385 | 0.03159 | down | -1.8476887 | *IL2RB* |
| ENSG00000008517 | 0.00010 | down | -3.5210583 | *IL32* |
| ENSG00000185291 | 0.00009 | down | -2.2058382 | *IL3RA* |
| ENSG00000160712 | 0.00001 | down | -2.981367 | *IL6R* |
| ENSG00000122641 | 0.00003 | down | -2.8250494 | *INHBA* |
| ENSG00000183016 | 0.00267 | down | -4.7934012 | *IQCA1P1* |
| ENSG00000106012 | 0.00287 | down | -1.5916728 | *IQCE* |
| ENSG00000229972 | 0.04568 | down | -1.7779574 | *IQCF3* |
| ENSG00000250588 | 0.00065 | down | -1.8623831 | *IQCJ-SCHIP1* |
| ENSG00000169047 | 0.00032 | down | -4.0425463 | *IRS1* |
| ENSG00000185950 | 0.00215 | down | -2.584069 | *IRS2* |
| ENSG00000176842 | 0.00008 | down | -3.5265348 | *IRX5* |
| ENSG00000159387 | 0.00004 | down | -2.378779 | *IRX6* |
| ENSG00000172183 | 0.00435 | down | -1.9149747 | *ISG20* |
| ENSG00000101230 | 0.00012 | down | -1.7743921 | *ISM1* |
| ENSG00000143127 | 0.00355 | down | -2.632066 | *ITGA10* |
| ENSG00000164171 | 0.00514 | down | -2.7159815 | *ITGA2* |
| ENSG00000005844 | 0.00251 | down | -2.8714578 | *ITGAL* |
| ENSG00000169896 | 0.00004 | down | -1.8121295 | *ITGAM* |
| ENSG00000140678 | 0.00004 | down | -3.4954891 | *ITGAX* |
| ENSG00000132470 | 0.02571 | down | -2.439592 | *ITGB4* |
| ENSG00000082781 | 0.00024 | down | -1.976942 | *ITGB5* |
| ENSG00000139626 | 0.00003 | down | -2.3709645 | *ITGB7* |
| ENSG00000105855 | 0.00227 | down | -1.7556475 | *ITGB8* |
| ENSG00000123243 | 0.02875 | down | -3.5181043 | *ITIH5* |
| ENSG00000096433 | 0.00234 | down | -1.689528 | *ITPR3* |
| ENSG00000188385 | 0.00006 | down | -4.123775 | *JAKMIP3* |
| ENSG00000104369 | 0.00246 | down | -2.2779534 | *JPH1* |
| ENSG00000177606 | 0.00004 | down | -1.8916165 | *JUN* |
| ENSG00000171223 | 0.00012 | down | -1.8592147 | *JUNB* |
| ENSG00000176595 | 0.00160 | down | -1.6489383 | *KBTBD11* |
| ENSG00000131398 | 0.00461 | down | -1.7806845 | *KCNC3* |
| ENSG00000102057 | 0.00266 | down | -1.659002 | *KCND1* |
| ENSG00000184408 | 0.00202 | down | -3.0190277 | *KCND2* |
| ENSG00000026559 | 0.03513 | down | -1.8044765 | *KCNG1* |
| ENSG00000089558 | 0.00363 | down | -1.8317502 | *KCNH4* |
| ENSG00000123700 | 0.00001 | down | -2.1344676 | *KCNJ2* |
| ENSG00000173338 | 0.00151 | down | -1.8837122 | *KCNK7* |
| ENSG00000156113 | 0.00078 | down | -2.4159398 | *KCNMA1* |
| ENSG00000105642 | 0.03449 | down | -2.4487119 | *KCNN1* |
| ENSG00000107147 | 0.00918 | down | -2.5934627 | *KCNT1* |
| ENSG00000132510 | 0.00009 | down | -1.7916666 | *KDM6B* |
| ENSG00000112232 | 0.00257 | down | -4.234459 | *KHDRBS2* |
| ENSG00000131773 | 0.00017 | down | -1.9901515 | *KHDRBS3* |
| ENSG00000103888 | 0.00470 | down | -2.5613291 | *KIAA1199* |
| ENSG00000110427 | 0.01438 | down | -3.2950842 | *KIAA1549L* |
| ENSG00000187164 | 0.00226 | down | -1.7882982 | *KIAA1598* |
| ENSG00000197077 | 0.00055 | down | -2.061873 | *KIAA1671* |
| ENSG00000130518 | 0.00003 | down | -2.0170774 | *KIAA1683* |
| ENSG00000116852 | 0.00001 | down | -2.1108856 | *KIF21B* |
| ENSG00000155980 | 0.00188 | down | -1.8126267 | *KIF5A* |
| ENSG00000126259 | 0.00323 | down | -5.630805 | *KIRREL2* |
| ENSG00000104892 | 0.01423 | down | -1.7694067 | *KLC3* |
| ENSG00000155090 | 0.00071 | down | -2.1176043 | *KLF10* |
| ENSG00000172059 | 0.00273 | down | -1.6447816 | *KLF11* |
| ENSG00000169926 | 0.00262 | down | -1.7259184 | *KLF13* |
| ENSG00000174595 | 0.02509 | down | -3.8037364 | *KLF14* |
| ENSG00000127528 | 0.02941 | down | -3.000503 | *KLF2* |
| ENSG00000136826 | 0.00000 | down | -3.7557225 | *KLF4* |
| ENSG00000102554 | 0.00171 | down | -2.8026109 | *KLF5* |
| ENSG00000067082 | 0.00003 | down | -1.9450849 | *KLF6* |
| ENSG00000119771 | 0.00001 | down | -2.294152 | *KLHL29* |
| ENSG00000168427 | 0.01052 | down | -6.6665854 | *KLHL30* |
| ENSG00000167759 | 0.00672 | down | -3.765585 | *KLK13* |
| ENSG00000129437 | 0.00673 | down | -3.001808 | *KLK14* |
| ENSG00000167751 | 0.00494 | down | -1.981639 | *KLK2* |
| ENSG00000164344 | 0.02677 | down | -1.8890371 | *KLKB1* |
| ENSG00000150045 | 0.00538 | down | -3.6995513 | *KLRF1* |
| ENSG00000171798 | 0.00519 | down | -4.5720406 | *KNDC1* |
| ENSG00000186395 | 0.00214 | down | -1.5568218 | *KRT10* |
| ENSG00000171401 | 0.00380 | down | -1.9979336 | *KRT13* |
| ENSG00000111057 | 0.01916 | down | -1.5586908 | *KRT18* |
| ENSG00000213943 | 0.03546 | down | -1.6526833 | *KRT18P17* |
| ENSG00000255815 | 0.04094 | down | -4.0331974 | *KRT8P11* |
| ENSG00000214659 | 0.00023 | down | -2.2276504 | *KRT8P26* |
| ENSG00000171403 | 0.00010 | down | -5.197409 | *KRT9* |
| ENSG00000198910 | 0.00105 | down | -2.1599913 | *L1CAM* |
| ENSG00000240563 | 0.00355 | down | -1.6476336 | *L1TD1* |
| ENSG00000089692 | 0.04417 | down | -3.0254147 | *LAG3* |
| ENSG00000053747 | 0.00047 | down | -2.2365222 | *LAMA3* |
| ENSG00000078081 | 0.00626 | down | -1.646731 | *LAMP3* |
| ENSG00000213398 | 0.00249 | down | -1.7634388 | *LCAT* |
| ENSG00000130164 | 0.00039 | down | -1.5907547 | *LDLR* |
| ENSG00000106003 | 0.00000 | down | -2.5017755 | *LFNG* |
| ENSG00000108679 | 0.00323 | down | -1.894149 | *LGALS3BP* |
| ENSG00000171747 | 0.02195 | down | -4.4276147 | *LGALS4* |
| ENSG00000168961 | 0.01297 | down | -1.7147475 | *LGALS9* |
| ENSG00000100600 | 0.00032 | down | -1.815295 | *LGMN* |
| ENSG00000139292 | 0.01403 | down | -2.723848 | *LGR5* |
| ENSG00000156959 | 0.00001 | down | -2.2367287 | *LHFPL4* |
| ENSG00000244482 | 0.03656 | down | -1.9689103 | *LILRA6* |
| ENSG00000186818 | 0.00785 | down | -5.3713565 | *LILRB4* |
| ENSG00000186152 | 0.00084 | down | -4.0929065 | *LILRP1* |
| ENSG00000149656 | 0.04627 | down | -1.6867313 | *LINC00266-1* |
| ENSG00000229645 | 0.02248 | down | -1.7982348 | *LINC00341* |
| ENSG00000215386 | 0.00315 | down | -1.9749432 | *LINC00478* |
| ENSG00000167117 | 0.02158 | down | -3.5534234 | *LINC00483* |
| ENSG00000260910 | 0.00036 | down | -3.7689102 | *LINC00565* |
| ENSG00000260941 | 0.00121 | down | -2.2372854 | *LINC00622* |
| ENSG00000263874 | 0.00810 | down | -2.6132905 | *LINC00672* |
| ENSG00000241163 | 0.00158 | down | -2.8666906 | *LINC00877* |
| ENSG00000258647 | 0.00038 | down | -2.8228137 | *LINC00930* |
| ENSG00000189238 | 0.00573 | down | -3.5010428 | *LINC00943* |
| ENSG00000249816 | 0.00241 | down | -1.7217262 | *LINC00964* |
| ENSG00000236700 | 0.01594 | down | -4.3319387 | *LINC01010* |
| ENSG00000253799 | 0.00916 | down | -4.0543513 | *LINC01030* |
| ENSG00000163898 | 0.01321 | down | -2.232361 | *LIPH* |
| ENSG00000140506 | 0.00016 | down | -3.6548839 | *LMAN1L* |
| ENSG00000175445 | 0.00001 | down | -1.9311948 | *LPL* |
| ENSG00000167419 | 0.02645 | down | -2.083804 | *LPO* |
| ENSG00000110031 | 0.00000 | down | -1.5509546 | *LPXN* |
| ENSG00000204583 | 0.00158 | down | -2.371765 | *LRCOL1* |
| ENSG00000144749 | 0.00105 | down | -1.567077 | *LRIG1* |
| ENSG00000160838 | 0.00096 | down | -3.728954 | *LRRC71* |
| ENSG00000171488 | 0.01093 | down | -1.6198207 | *LRRC8C* |
| ENSG00000181016 | 0.00197 | down | -1.7903163 | *LSMEM1* |
| ENSG00000119681 | 0.00044 | down | -2.3543692 | *LTBP2* |
| ENSG00000168056 | 0.01881 | down | -1.7428688 | *LTBP3* |
| ENSG00000139329 | 0.00796 | down | -3.2471862 | *LUM* |
| ENSG00000102897 | 0.00103 | down | -1.5777762 | *LYRM1* |
| ENSG00000088899 | 0.01135 | down | -1.7142266 | *LZTS3* |
| ENSG00000182759 | 0.00049 | down | -2.3245935 | *MAFA* |
| ENSG00000204103 | 0.00843 | down | -2.0392184 | *MAFB* |
| ENSG00000185022 | 0.00005 | down | -2.2198293 | *MAFF* |
| ENSG00000198517 | 0.00065 | down | -1.582342 | *MAFK* |
| ENSG00000184750 | 0.00056 | down | -3.2614615 | *MAGEA2* |
| ENSG00000183305 | 0.00056 | down | -3.3476655 | *MAGEA2B* |
| ENSG00000221867 | 0.00007 | down | -2.6855228 | *MAGEA3* |
| ENSG00000197172 | 0.00045 | down | -2.3831728 | *MAGEA6* |
| ENSG00000177689 | 0.00000 | down | -5.092935 | *MAGEB10* |
| ENSG00000046774 | 0.00029 | down | -1.6782132 | *MAGEC2* |
| ENSG00000179222 | 0.00100 | down | -1.6761427 | *MAGED1* |
| ENSG00000154545 | 0.00154 | down | -2.8252056 | *MAGED4* |
| ENSG00000187243 | 0.00098 | down | -2.9019349 | *MAGED4B* |
| ENSG00000139625 | 0.01767 | down | -1.6366726 | *MAP3K12* |
| ENSG00000142733 | 0.00005 | down | -1.8093557 | *MAP3K6* |
| ENSG00000006432 | 0.00729 | down | -1.8116772 | *MAP3K9* |
| ENSG00000171533 | 0.03879 | down | -2.8155515 | *MAP6* |
| ENSG00000181085 | 0.00025 | down | -4.0297885 | *MAPK15* |
| ENSG00000141639 | 0.00001 | down | -2.5333533 | *MAPK4* |
| ENSG00000121653 | 0.00581 | down | -2.303472 | *MAPK8IP1* |
| ENSG00000186868 | 0.01503 | down | -2.131765 | *MAPT* |
| ENSG00000155130 | 0.00032 | down | -1.5222194 | *MARCKS* |
| ENSG00000105613 | 0.01915 | down | -1.5773225 | *MAST1* |
| ENSG00000180611 | 0.00078 | down | -2.146728 | *MB21D2* |
| ENSG00000258839 | 0.00721 | down | -1.8722508 | *MC1R* |
| ENSG00000101977 | 0.04108 | down | -2.7423651 | *MCF2* |
| ENSG00000224328 | 0.02316 | down | -1.7710826 | *MDC1-AS1* |
| ENSG00000112139 | 0.00008 | down | -1.7945971 | *MDGA1* |
| ENSG00000229111 | 0.00757 | down | -2.4405146 | *MED4-AS1* |
| ENSG00000105419 | 0.00837 | down | -1.8424661 | *MEIS3* |
| ENSG00000139780 | 0.00055 | down | -2.0042887 | *METTL21C* |
| ENSG00000181588 | 0.04097 | down | -1.6060948 | *MEX3D* |
| ENSG00000140545 | 0.01989 | down | -1.5151625 | *MFGE8* |
| ENSG00000135596 | 0.00089 | down | -1.5006123 | *MICAL1* |
| ENSG00000238123 | 0.01845 | down | -1.8850952 | *MID1IP1-AS1* |
| ENSG00000167470 | 0.00211 | down | -2.3026297 | *MIDN* |
| ENSG00000264462 | 0.01308 | down | -2.4255648 | *MIR3648* |
| ENSG00000264063 | 0.00067 | down | -2.8377798 | *MIR3687* |
| ENSG00000264021 | 0.03671 | down | -2.2660408 | *MIR3917* |
| ENSG00000202566 | 0.00768 | down | -1.7275649 | *MIR421* |
| ENSG00000227195 | 0.00308 | down | -2.359946 | *MIR663A* |
| ENSG00000221288 | 0.04286 | down | -4.028077 | *MIR663B* |
| ENSG00000227154 | 0.00008 | down | -3.854431 | *MKRNP2* |
| ENSG00000130396 | 0.00456 | down | -1.8548421 | *MLLT4* |
| ENSG00000009950 | 0.00048 | down | -3.2568266 | *MLXIPL* |
| ENSG00000166670 | 0.00084 | down | -1.8507856 | *MMP10* |
| ENSG00000173269 | 0.02255 | down | -3.743429 | *MMRN2* |
| ENSG00000169184 | 0.00021 | down | -2.13029 | *MN1* |
| ENSG00000005381 | 0.00468 | down | -3.6837676 | *MPO* |
| ENSG00000160588 | 0.00037 | down | -1.7453797 | *MPZL3* |
| ENSG00000226807 | 0.00464 | down | -2.236675 | *MROH5* |
| ENSG00000188981 | 0.03280 | down | -1.5803701 | *MSANTD1* |
| ENSG00000178860 | 0.00007 | down | -2.6122918 | *MSC* |
| ENSG00000038945 | 0.00090 | down | -1.6443635 | *MSR1* |
| ENSG00000162840 | 0.01593 | down | -1.8816721 | *MT2P1* |
| ENSG00000014914 | 0.00126 | down | -2.7864482 | *MTMR11* |
| ENSG00000231888 | 0.00000 | down | -2.021806 | *MTND5P15* |
| ENSG00000179141 | 0.00044 | down | -2.6655834 | *MTUS2-AS1* |
| ENSG00000185499 | 0.00031 | down | -1.9928592 | *MUC1* |
| ENSG00000173702 | 0.00291 | down | -4.065919 | *MUC13* |
| ENSG00000145113 | 0.04925 | down | -4.2940254 | *MUC4* |
| ENSG00000184956 | 0.00251 | down | -3.262778 | *MUC6* |
| ENSG00000179820 | 0.00011 | down | -1.9364222 | *MYADM* |
| ENSG00000007944 | 0.00002 | down | -1.7005342 | *MYLIP* |
| ENSG00000137474 | 0.01659 | down | -1.8423733 | *MYO7A* |
| ENSG00000145911 | 0.00001 | down | -1.9353294 | *N4BP3* |
| ENSG00000136274 | 0.04080 | down | -2.1612911 | *NACAD* |
| ENSG00000067798 | 0.02168 | down | -2.90441 | *NAV3* |
| ENSG00000240108 | 0.01292 | down | -2.948156 | *NCOR1P1* |
| ENSG00000173376 | 0.00068 | down | -5.29272 | *NDNF* |
| ENSG00000104419 | 0.00000 | down | -2.2512062 | *NDRG1* |
| ENSG00000138653 | 0.03328 | down | -1.535545 | *NDST4* |
| ENSG00000185633 | 0.00201 | down | -2.0299308 | *NDUFA4L2* |
| ENSG00000163491 | 0.01191 | down | -3.1994483 | *NEK10* |
| ENSG00000204099 | 0.00943 | down | -2.4601765 | *NEU4* |
| ENSG00000131196 | 0.00366 | down | -1.8057102 | *NFATC1* |
| ENSG00000165030 | 0.00004 | down | -1.9373459 | *NFIL3* |
| ENSG00000170448 | 0.00220 | down | -1.5068263 | *NFXL1* |
| ENSG00000064300 | 0.01065 | down | -1.6396792 | *NGFR* |
| ENSG00000116962 | 0.00060 | down | -3.093759 | *NID1* |
| ENSG00000131669 | 0.00000 | down | -1.6771021 | *NINJ1* |
| ENSG00000163293 | 0.00438 | down | -2.0113068 | *NIPAL1* |
| ENSG00000188580 | 0.02501 | down | -2.2549143 | *NKAIN2* |
| ENSG00000165066 | 0.00896 | down | -4.3295965 | *NKX6-3* |
| ENSG00000167984 | 0.00360 | down | -1.7365674 | *NLRC3* |
| ENSG00000091592 | 0.00033 | down | -2.030258 | *NLRP1* |
| ENSG00000171487 | 0.00069 | down | -3.2673943 | *NLRP5* |
| ENSG00000174885 | 0.00827 | down | -3.3162305 | *NLRP6* |
| ENSG00000185792 | 0.01703 | down | -1.9913381 | *NLRP9* |
| ENSG00000109255 | 0.02649 | down | -1.6828351 | *NMU* |
| ENSG00000167207 | 0.00009 | down | -1.6396123 | *NOD2* |
| ENSG00000140939 | 0.01247 | down | -1.6213787 | *NOL3* |
| ENSG00000237740 | 0.00069 | down | -3.2064269 | *NPAP1P3* |
| ENSG00000141458 | 0.00161 | down | -1.5705383 | *NPC1* |
| ENSG00000015520 | 0.00107 | down | -2.3107457 | *NPC1L1* |
| ENSG00000183426 | 0.00537 | down | -1.5409849 | *NPIPA1* |
| ENSG00000214940 | 0.00120 | down | -1.6136148 | *NPIPA8* |
| ENSG00000198156 | 0.00015 | down | -1.8076787 | *NPIPB6* |
| ENSG00000106236 | 0.03197 | down | -3.104201 | *NPTX2* |
| ENSG00000123358 | 0.00019 | down | -2.7720935 | *NR4A1* |
| ENSG00000153234 | 0.00027 | down | -1.8855348 | *NR4A2* |
| ENSG00000119508 | 0.00168 | down | -2.6271062 | *NR4A3* |
| ENSG00000091129 | 0.00249 | down | -1.6495372 | *NRCAM* |
| ENSG00000157168 | 0.01416 | down | -1.9396739 | *NRG1* |
| ENSG00000170091 | 0.01347 | down | -2.4499643 | *NSG2* |
| ENSG00000065320 | 0.00299 | down | -2.1486464 | *NTN1* |
| ENSG00000105245 | 0.00189 | down | -1.6079483 | *NUMBL* |
| ENSG00000126883 | 0.00398 | down | -1.5110054 | *NUP214* |
| ENSG00000167693 | 0.00000 | down | -6.296502 | *NXN* |
| ENSG00000154358 | 0.00303 | down | -1.7618972 | *OBSCN* |
| ENSG00000177947 | 0.00065 | down | -3.8600926 | *ODF3* |
| ENSG00000152463 | 0.00052 | down | -1.9584303 | *OLAH* |
| ENSG00000178814 | 0.00678 | down | -2.2730336 | *OPLAH* |
| ENSG00000054277 | 0.00322 | down | -1.5756953 | *OPN3* |
| ENSG00000116329 | 0.00027 | down | -2.7063916 | *OPRD1* |
| ENSG00000196071 | 0.01672 | down | -2.4814074 | *OR2L13* |
| ENSG00000217315 | 0.00014 | down | -3.410354 | *OR2W2P* |
| ENSG00000186092 | 0.02653 | down | -1.536717 | *OR4F5* |
| ENSG00000164920 | 0.01936 | down | -2.036323 | *OSR2* |
| ENSG00000115155 | 0.01564 | down | -4.047392 | *OTOF* |
| ENSG00000188162 | 0.02720 | down | -3.5169437 | *OTOG* |
| ENSG00000165312 | 0.00007 | down | -4.11907 | *OTUD1* |
| ENSG00000085465 | 0.02144 | down | -1.581418 | *OVGP1* |
| ENSG00000172818 | 0.00000 | down | -3.9285073 | *OVOL1* |
| ENSG00000101104 | 0.00604 | down | -1.5676887 | *PABPC1L* |
| ENSG00000186288 | 0.01099 | down | -3.0501537 | *PABPC1L2A* |
| ENSG00000184388 | 0.02658 | down | -2.6645548 | *PABPC1L2B* |
| ENSG00000124507 | 0.00193 | down | -2.439388 | *PACSIN1* |
| ENSG00000076641 | 0.02401 | down | -1.8570324 | *PAG1* |
| ENSG00000160781 | 0.00505 | down | -2.207411 | *PAQR6* |
| ENSG00000177425 | 0.00005 | down | -1.5448352 | *PAWR* |
| ENSG00000105717 | 0.00011 | down | -1.6471323 | *PBX4* |
| ENSG00000102290 | 0.01441 | down | -3.019028 | *PCDH11X* |
| ENSG00000261934 | 0.03765 | down | -3.4526641 | *PCDHGA9* |
| ENSG00000254122 | 0.00272 | down | -2.687935 | *PCDHGB7* |
| ENSG00000240184 | 0.03519 | down | -2.5693865 | *PCDHGC3* |
| ENSG00000242419 | 0.00493 | down | -2.9726608 | *PCDHGC4* |
| ENSG00000179715 | 0.00009 | down | -1.7357405 | *PCED1B* |
| ENSG00000247774 | 0.00016 | down | -2.507641 | *PCED1B-AS1* |
| ENSG00000174788 | 0.03275 | down | -1.9641114 | *PCP2* |
| ENSG00000105650 | 0.00098 | down | -3.9582918 | *PDE4C* |
| ENSG00000185527 | 0.03358 | down | -3.4837782 | *PDE6G* |
| ENSG00000197461 | 0.00062 | down | -2.7160487 | *PDGFA* |
| ENSG00000004799 | 0.00011 | down | -2.9817564 | *PDK4* |
| ENSG00000133401 | 0.01996 | down | -1.7841121 | *PDZD2* |
| ENSG00000179094 | 0.00212 | down | -2.214898 | *PER1* |
| ENSG00000112378 | 0.00006 | down | -1.5682812 | *PERP* |
| ENSG00000213997 | 0.00687 | down | -4.1172686 | *PGAM1P7* |
| ENSG00000177614 | 0.00070 | down | -2.5258577 | *PGBD5* |
| ENSG00000082175 | 0.01159 | down | -4.445012 | *PGR* |
| ENSG00000139289 | 0.00033 | down | -1.7448702 | *PHLDA1* |
| ENSG00000081913 | 0.00223 | down | -1.5583044 | *PHLPP1* |
| ENSG00000164530 | 0.00058 | down | -2.6033387 | *PI16* |
| ENSG00000171608 | 0.00008 | down | -1.5577124 | *PIK3CD* |
| ENSG00000100100 | 0.00010 | down | -2.7972343 | *PIK3IP1* |
| ENSG00000091622 | 0.00065 | down | -3.49705 | *PITPNM3* |
| ENSG00000158683 | 0.01930 | down | -3.1591275 | *PKD1L1* |
| ENSG00000187008 | 0.02165 | down | -3.0258465 | *PKD1L3* |
| ENSG00000143627 | 0.00072 | down | -3.866837 | *PKLR* |
| ENSG00000184381 | 0.00039 | down | -1.7999371 | *PLA2G6* |
| ENSG00000153246 | 0.02330 | down | -2.4205563 | *PLA2R1* |
| ENSG00000178209 | 0.00120 | down | -1.8107728 | *PLEC* |
| ENSG00000107679 | 0.00561 | down | -2.0510283 | *PLEKHA1* |
| ENSG00000143850 | 0.00002 | down | -2.105418 | *PLEKHA6* |
| ENSG00000120278 | 0.03320 | down | -1.7531866 | *PLEKHG1* |
| ENSG00000152527 | 0.01960 | down | -2.0843267 | *PLEKHH2* |
| ENSG00000068137 | 0.00303 | down | -2.1916492 | *PLEKHH3* |
| ENSG00000148735 | 0.00045 | down | -3.2897599 | *PLEKHS1* |
| ENSG00000183281 | 0.01188 | down | -1.8889211 | *PLGLB1* |
| ENSG00000145632 | 0.00000 | down | -1.6720638 | *PLK2* |
| ENSG00000198523 | 0.00069 | down | -2.4493647 | *PLN* |
| ENSG00000146281 | 0.00020 | down | -2.1576483 | *PM20D2* |
| ENSG00000141682 | 0.00018 | down | -2.0427132 | *PMAIP1* |
| ENSG00000130822 | 0.00676 | down | -2.9807858 | *PNCK* |
| ENSG00000130653 | 0.00005 | down | -2.3679528 | *PNPLA7* |
| ENSG00000146278 | 0.00005 | down | -1.6806945 | *PNRC1* |
| ENSG00000128567 | 0.01670 | down | -1.5629051 | *PODXL* |
| ENSG00000028277 | 0.00003 | down | -1.5441151 | *POU2F2* |
| ENSG00000185668 | 0.00591 | down | -2.8539357 | *POU3F1* |
| ENSG00000183977 | 0.03596 | down | -1.639584 | *PP2D1* |
| ENSG00000162407 | 0.00028 | down | -2.8879278 | *PPAP2B* |
| ENSG00000141934 | 0.00573 | down | -2.4083283 | *PPAP2C* |
| ENSG00000112033 | 0.00056 | down | -1.5710592 | *PPARD* |
| ENSG00000156194 | 0.02381 | down | -3.4522762 | *PPEF2* |
| ENSG00000143847 | 0.01392 | down | -2.3391228 | *PPFIA4* |
| ENSG00000118898 | 0.00105 | down | -2.2069116 | *PPL* |
| ENSG00000204569 | 0.00080 | down | -1.6483173 | *PPP1R10* |
| ENSG00000198729 | 0.00466 | down | -2.1340044 | *PPP1R14C* |
| ENSG00000124224 | 0.00008 | down | -2.7279196 | *PPP4R1L* |
| ENSG00000057657 | 0.00000 | down | -2.6983109 | *PRDM1* |
| ENSG00000235816 | 0.00001 | down | -2.9271574 | *PRELID1P3* |
| ENSG00000180644 | 0.00016 | down | -3.3834488 | *PRF1* |
| ENSG00000162409 | 0.00003 | down | -2.2092328 | *PRKAA2* |
| ENSG00000154229 | 0.01887 | down | -1.5843481 | *PRKCA* |
| ENSG00000126583 | 0.00019 | down | -3.9048746 | *PRKCG* |
| ENSG00000117707 | 0.00197 | down | -2.2455776 | *PROX1* |
| ENSG00000163704 | 0.00252 | down | -1.6696615 | *PRRT3* |
| ENSG00000237412 | 0.01426 | down | -2.1756518 | *PRSS56* |
| ENSG00000105227 | 0.00087 | down | -2.3943758 | *PRX* |
| ENSG00000146005 | 0.00219 | down | -1.8953648 | *PSD2* |
| ENSG00000140368 | 0.00399 | down | -2.500423 | *PSTPIP1* |
| ENSG00000185920 | 0.00638 | down | -1.5552005 | *PTCH1* |
| ENSG00000106853 | 0.00026 | down | -1.6968876 | *PTGR1* |
| ENSG00000070778 | 0.00456 | down | -2.239918 | *PTPN21* |
| ENSG00000127329 | 0.01613 | down | -1.7501615 | *PTPRB* |
| ENSG00000080031 | 0.00284 | down | -1.7545261 | *PTPRH* |
| ENSG00000152894 | 0.04939 | down | -1.5624702 | *PTPRK* |
| ENSG00000153233 | 0.00776 | down | -2.1019926 | *PTPRR* |
| ENSG00000105426 | 0.02168 | down | -1.8364433 | *PTPRS* |
| ENSG00000068976 | 0.00011 | down | -3.1816373 | *PYGM* |
| ENSG00000237575 | 0.01516 | down | -2.0271003 | *PYY2* |
| ENSG00000156675 | 0.00046 | down | -1.621969 | *RAB11FIP1* |
| ENSG00000169213 | 0.00023 | down | -1.7927262 | *RAB3B* |
| ENSG00000164080 | 0.00048 | down | -1.5371141 | *RAD54L2* |
| ENSG00000155918 | 0.03295 | down | -2.8802125 | *RAET1L* |
| ENSG00000039560 | 0.02950 | down | -1.5172079 | *RAI14* |
| ENSG00000131831 | 0.00175 | down | -2.3049178 | *RAI2* |
| ENSG00000116191 | 0.00005 | down | -2.7129414 | *RALGPS2* |
| ENSG00000132329 | 0.02395 | down | -2.5246887 | *RAMP1* |
| ENSG00000132359 | 0.00040 | down | -1.742166 | *RAP1GAP2* |
| ENSG00000131759 | 0.00202 | down | -1.8129826 | *RARA* |
| ENSG00000172819 | 0.00276 | down | -1.8876122 | *RARG* |
| ENSG00000075391 | 0.00198 | down | -2.1410453 | *RASAL2* |
| ENSG00000108551 | 0.00014 | down | -2.4305906 | *RASD1* |
| ENSG00000173976 | 0.00000 | down | -2.093277 | *RAX2* |
| ENSG00000112183 | 0.00011 | down | -5.0760646 | *RBM24* |
| ENSG00000177483 | 0.01085 | down | -1.702684 | *RBM44* |
| ENSG00000163694 | 0.00083 | down | -2.0255868 | *RBM47* |
| ENSG00000242875 | 0.00014 | down | -1.9394567 | *RBMY1B* |
| ENSG00000244395 | 0.02606 | down | -1.6775302 | *RBMY1D* |
| ENSG00000242389 | 0.00958 | down | -1.8276724 | *RBMY1E* |
| ENSG00000169800 | 0.00581 | down | -1.7551615 | *RBMY1F* |
| ENSG00000169811 | 0.00040 | down | -1.7151484 | *RBMY1HP* |
| ENSG00000226941 | 0.00055 | down | -1.9606866 | *RBMY1J* |
| ENSG00000117602 | 0.00417 | down | -1.716313 | *RCAN3* |
| ENSG00000174136 | 0.00609 | down | -1.7520369 | *RGMB* |
| ENSG00000090104 | 0.00004 | down | -4.254351 | *RGS1* |
| ENSG00000076344 | 0.02371 | down | -3.499066 | *RGS11* |
| ENSG00000143333 | 0.00396 | down | -1.6662229 | *RGS16* |
| ENSG00000116741 | 0.00034 | down | -2.070386 | *RGS2* |
| ENSG00000140519 | 0.02778 | down | -3.298706 | *RHCG* |
| ENSG00000143878 | 0.00004 | down | -2.5616124 | *RHOB* |
| ENSG00000131941 | 0.00001 | down | -2.3138418 | *RHPN2* |
| ENSG00000196934 | 0.04012 | down | -2.507674 | *RIMBP3B* |
| ENSG00000183246 | 0.02703 | down | -2.5394197 | *RIMBP3C* |
| ENSG00000202058 | 0.01636 | down | -1.8306129 | *RN7SKP80* |
| ENSG00000239884 | 0.01647 | down | -2.234365 | *RN7SL608P* |
| ENSG00000265577 | 0.01917 | down | -1.8509831 | *RN7SL9P* |
| ENSG00000172602 | 0.00215 | down | -1.8990136 | *RND1* |
| ENSG00000115963 | 0.00303 | down | -1.8057892 | *RND3* |
| ENSG00000176641 | 0.01462 | down | -1.7308922 | *RNF152* |
| ENSG00000116514 | 0.00001 | down | -1.576795 | *RNF19B* |
| ENSG00000173821 | 0.03802 | down | -1.5723991 | *RNF213* |
| ENSG00000108375 | 0.00004 | down | -1.5949969 | *RNF43* |
| ENSG00000185008 | 0.00031 | down | -1.5107934 | *ROBO2* |
| ENSG00000240370 | 0.00701 | down | -1.728608 | *RPL13P5* |
| ENSG00000225246 | 0.03746 | down | -5.6821036 | *RPS2P1* |
| ENSG00000243609 | 0.01975 | down | -2.0487256 | *RPS2P44* |
| ENSG00000108309 | 0.00352 | down | -2.0161996 | *RUNDC3A* |
| ENSG00000105784 | 0.00287 | down | -1.7649753 | *RUNDC3B* |
| ENSG00000124813 | 0.00086 | down | -2.0860333 | *RUNX2* |
| ENSG00000198853 | 0.00551 | down | -1.7614477 | *RUSC2* |
| ENSG00000173080 | 0.00610 | down | -3.3267114 | *RXFP4* |
| ENSG00000196218 | 0.03308 | down | -1.7992129 | *RYR1* |
| ENSG00000170989 | 0.02241 | down | -1.6187377 | *S1PR1* |
| ENSG00000213694 | 0.00013 | down | -1.9238597 | *S1PR3* |
| ENSG00000181433 | 0.00016 | down | -1.8082262 | *SAGE1* |
| ENSG00000165821 | 0.00265 | down | -2.9424112 | *SALL2* |
| ENSG00000203727 | 0.00039 | down | -2.1286786 | *SAMD5* |
| ENSG00000004139 | 0.00286 | down | -1.6296924 | *SARM1* |
| ENSG00000122122 | 0.00083 | down | -3.2184343 | *SASH3* |
| ENSG00000130066 | 0.00031 | down | -1.5997657 | *SAT1* |
| ENSG00000119042 | 0.00013 | down | -2.2586179 | *SATB2* |
| ENSG00000187550 | 0.02833 | down | -2.4208 | *SBK2* |
| ENSG00000189001 | 0.04752 | down | -2.6413147 | *SBSN* |
| ENSG00000252808 | 0.04678 | down | -2.512596 | *SCARNA4* |
| ENSG00000006747 | 0.00000 | down | -4.585397 | *SCIN* |
| ENSG00000007314 | 0.00001 | down | -3.1818678 | *SCN4A* |
| ENSG00000159307 | 0.00314 | down | -1.9708501 | *SCUBE1* |
| ENSG00000175356 | 0.02442 | down | -2.5107458 | *SCUBE2* |
| ENSG00000110876 | 0.02575 | down | -1.6387242 | *SELPLG* |
| ENSG00000012171 | 0.00119 | down | -3.0328004 | *SEMA3B* |
| ENSG00000001617 | 0.03071 | down | -3.6823626 | *SEMA3F* |
| ENSG00000196189 | 0.00522 | down | -2.6938834 | *SEMA4A* |
| ENSG00000092421 | 0.00044 | down | -1.5267073 | *SEMA6A* |
| ENSG00000170542 | 0.00260 | down | -1.6049857 | *SERPINB9* |
| ENSG00000163069 | 0.00088 | down | -1.7393526 | *SGCB* |
| ENSG00000185053 | 0.04435 | down | -3.1084738 | *SGCZ* |
| ENSG00000118515 | 0.00000 | down | -2.3535304 | *SGK1* |
| ENSG00000164023 | 0.00176 | down | -2.3874598 | *SGMS2* |
| ENSG00000166224 | 0.00181 | down | -1.7795402 | *SGPL1* |
| ENSG00000125731 | 0.00034 | down | -1.9058899 | *SH2D3A* |
| ENSG00000107295 | 0.00296 | down | -3.951946 | *SH3GL2* |
| ENSG00000146950 | 0.01597 | down | -4.792408 | *SHROOM2* |
| ENSG00000179213 | 0.00025 | down | -1.9068358 | *SIGLECL1* |
| ENSG00000198053 | 0.01176 | down | -1.7230719 | *SIRPA* |
| ENSG00000221955 | 0.00227 | down | -2.1323786 | *SLC12A8* |
| ENSG00000164707 | 0.03639 | down | -1.6763684 | *SLC13A4* |
| ENSG00000141485 | 0.01715 | down | -2.5837896 | *SLC13A5* |
| ENSG00000141526 | 0.00059 | down | -1.7792284 | *SLC16A3* |
| ENSG00000197847 | 0.00076 | down | -1.7800177 | *SLC22A20* |
| ENSG00000137266 | 0.00002 | down | -1.8943655 | *SLC22A23* |
| ENSG00000183048 | 0.00910 | down | -1.6364712 | *SLC25A10* |
| ENSG00000182902 | 0.01914 | down | -1.9775159 | *SLC25A18* |
| ENSG00000146411 | 0.00712 | down | -1.7757976 | *SLC2A12* |
| ENSG00000109667 | 0.00105 | down | -2.1802552 | *SLC2A9* |
| ENSG00000170385 | 0.00509 | down | -2.0695496 | *SLC30A1* |
| ENSG00000188338 | 0.00151 | down | -3.250897 | *SLC38A3* |
| ENSG00000076351 | 0.01696 | down | -1.8770777 | *SLC46A1* |
| ENSG00000010379 | 0.03342 | down | -1.9095339 | *SLC6A13* |
| ENSG00000131389 | 0.00795 | down | -1.5996983 | *SLC6A6* |
| ENSG00000118160 | 0.01564 | down | -2.953025 | *SLC8A2* |
| ENSG00000065923 | 0.00126 | down | -2.5275176 | *SLC9A7* |
| ENSG00000172716 | 0.01580 | down | -5.923024 | *SLFN11* |
| ENSG00000145147 | 0.00641 | down | -2.4412751 | *SLIT2* |
| ENSG00000178235 | 0.00103 | down | -2.4025214 | *SLITRK1* |
| ENSG00000184564 | 0.00000 | down | -1.8411411 | *SLITRK6* |
| ENSG00000224960 | 0.00018 | down | -2.0142066 | *SMEK3P* |
| ENSG00000163683 | 0.00186 | down | -1.9673836 | *SMIM14* |
| ENSG00000088826 | 0.00127 | down | -1.7451638 | *SMOX* |
| ENSG00000251806 | 0.00242 | down | -2.3981946 | *SNORD119* |
| ENSG00000264940 | 0.02677 | down | -1.7168567 | *SNORD3C* |
| ENSG00000206602 | 0.00307 | down | -3.633463 | *SNORD58A* |
| ENSG00000271982 | 0.00000 | down | -36.831165 | *SNORD58B* |
| ENSG00000207031 | 0.00868 | down | -3.6994348 | *SNORD59A* |
| ENSG00000184985 | 0.02305 | down | -3.307376 | *SORCS2* |
| ENSG00000187808 | 0.00765 | down | -2.525113 | *SOWAHD* |
| ENSG00000143842 | 0.00475 | down | -1.8902401 | *SOX13* |
| ENSG00000110693 | 0.00047 | down | -1.6673865 | *SOX6* |
| ENSG00000005513 | 0.01664 | down | -3.1422377 | *SOX8* |
| ENSG00000167182 | 0.01787 | down | -1.559812 | *SP2* |
| ENSG00000189120 | 0.00103 | down | -3.148691 | *SP6* |
| ENSG00000104450 | 0.02039 | down | -1.5922496 | *SPAG1* |
| ENSG00000061656 | 0.03754 | down | -1.8682553 | *SPAG4* |
| ENSG00000269404 | 0.00436 | down | -4.1975017 | *SPIB* |
| ENSG00000182557 | 0.00826 | down | -2.1633997 | *SPNS3* |
| ENSG00000159674 | 0.00009 | down | -2.367387 | *SPON2* |
| ENSG00000198369 | 0.00269 | down | -1.761438 | *SPRED2* |
| ENSG00000187678 | 0.00010 | down | -2.4204733 | *SPRY4* |
| ENSG00000171621 | 0.01080 | down | -1.7436278 | *SPSB1* |
| ENSG00000115306 | 0.00039 | down | -1.9771186 | *SPTBN1* |
| ENSG00000137877 | 0.00011 | down | -2.5266004 | *SPTBN5* |
| ENSG00000197122 | 0.00092 | down | -1.6454005 | *SRC* |
| ENSG00000049319 | 0.00607 | down | -1.7894796 | *SRD5A2* |
| ENSG00000084112 | 0.03967 | down | -1.5889548 | *SSH1* |
| ENSG00000197558 | 0.00155 | down | -2.7238126 | *SSPO* |
| ENSG00000183473 | 0.00002 | down | -16.808847 | *SSTR3* |
| ENSG00000064225 | 0.02740 | down | -2.259136 | *ST3GAL6* |
| ENSG00000140557 | 0.00003 | down | -2.8868194 | *ST8SIA2* |
| ENSG00000099365 | 0.04969 | down | -1.7072221 | *STX1B* |
| ENSG00000197321 | 0.01850 | down | -1.6064242 | *SVIL* |
| ENSG00000008056 | 0.00054 | down | -3.139445 | *SYN1* |
| ENSG00000131018 | 0.00098 | down | -2.176644 | *SYNE1* |
| ENSG00000176438 | 0.00219 | down | -1.6488603 | *SYNE3* |
| ENSG00000078269 | 0.02123 | down | -1.5452436 | *SYNJ2* |
| ENSG00000171992 | 0.00786 | down | -1.789574 | *SYNPO* |
| ENSG00000132872 | 0.00255 | down | -2.9682462 | *SYT4* |
| ENSG00000170743 | 0.01412 | down | -2.2522256 | *SYT9* |
| ENSG00000149591 | 0.00632 | down | -1.9585751 | *TAGLN* |
| ENSG00000112837 | 0.00004 | down | -1.524504 | *TBX18* |
| ENSG00000121075 | 0.00089 | down | -1.7076471 | *TBX4* |
| ENSG00000148737 | 0.00366 | down | -1.9549199 | *TCF7L2* |
| ENSG00000166046 | 0.00313 | down | -2.6149154 | *TCP11L2* |
| ENSG00000146221 | 0.00671 | down | -2.4465666 | *TCTE1* |
| ENSG00000168778 | 0.00365 | down | -1.6396295 | *TCTN2* |
| ENSG00000092850 | 0.02000 | down | -2.2703257 | *TEKT2* |
| ENSG00000135269 | 0.00000 | down | -1.9302026 | *TES* |
| ENSG00000070759 | 0.00388 | down | -1.5849065 | *TESK2* |
| ENSG00000121101 | 0.00040 | down | -1.9612495 | *TEX14* |
| ENSG00000087510 | 0.00070 | down | -4.195429 | *TFAP2C* |
| ENSG00000112561 | 0.00331 | down | -1.6036173 | *TFEB* |
| ENSG00000042832 | 0.00443 | down | -1.5301886 | *TG* |
| ENSG00000163235 | 0.00046 | down | -5.649801 | *TGFA* |
| ENSG00000069702 | 0.00025 | down | -1.9657006 | *TGFBR3* |
| ENSG00000178726 | 0.00004 | down | -1.6712162 | *THBD* |
| ENSG00000173825 | 0.00120 | down | -2.7214208 | *TIGD3* |
| ENSG00000223573 | 0.00018 | down | -1.5535718 | *TINCR* |
| ENSG00000119139 | 0.00018 | down | -1.5999748 | *TJP2* |
| ENSG00000185561 | 0.00992 | down | -1.8220396 | *TLCD2* |
| ENSG00000167608 | 0.01024 | down | -2.350393 | *TMC4* |
| ENSG00000057704 | 0.03647 | down | -1.5897108 | *TMCC3* |
| ENSG00000164855 | 0.01430 | down | -1.7812263 | *TMEM184A* |
| ENSG00000182796 | 0.02964 | down | -1.6130737 | *TMEM198B* |
| ENSG00000253304 | 0.00000 | down | -16.459349 | *TMEM200B* |
| ENSG00000184497 | 0.03111 | down | -1.6761485 | *TMEM255B* |
| ENSG00000072954 | 0.01128 | down | -2.3899996 | *TMEM38A* |
| ENSG00000137747 | 0.01047 | down | -2.4110322 | *TMPRSS13* |
| ENSG00000154646 | 0.00005 | down | -2.1225426 | *TMPRSS15* |
| ENSG00000178297 | 0.00171 | down | -2.0258577 | *TMPRSS9* |
| ENSG00000141655 | 0.01284 | down | -1.6596932 | *TNFRSF11A* |
| ENSG00000121858 | 0.00000 | down | -1.8540947 | *TNFSF10* |
| ENSG00000125657 | 0.00447 | down | -1.6225456 | *TNFSF9* |
| ENSG00000131746 | 0.00005 | down | -2.3069763 | *TNS4* |
| ENSG00000124191 | 0.00009 | down | -3.4384987 | *TOX2* |
| ENSG00000078804 | 0.00129 | down | -1.7953211 | *TP53INP2* |
| ENSG00000186854 | 0.00325 | down | -3.1462297 | *TRABD2A* |
| ENSG00000211772 | 0.00954 | down | -2.2320318 | *TRBC2* |
| ENSG00000211767 | 0.00255 | down | -1.775426 | *TRBJ2-3* |
| ENSG00000211768 | 0.00220 | down | -2.8840272 | *TRBJ2-4* |
| ENSG00000231165 | 0.00687 | down | -3.1639373 | *TRBV26OR9-2* |
| ENSG00000173334 | 0.00003 | down | -2.719085 | *TRIB1* |
| ENSG00000109654 | 0.00235 | down | -5.2176723 | *TRIM2* |
| ENSG00000152503 | 0.00051 | down | -2.295663 | *TRIM36* |
| ENSG00000204614 | 0.00232 | down | -3.6182787 | *TRIM40* |
| ENSG00000206557 | 0.00050 | down | -3.7758908 | *TRIM71* |
| ENSG00000177238 | 0.00151 | down | -3.4852004 | *TRIM72* |
| ENSG00000248211 | 0.00367 | down | -2.807477 | *TRPC7-AS1* |
| ENSG00000165125 | 0.00649 | down | -2.6152818 | *TRPV6* |
| ENSG00000196428 | 0.02085 | down | -1.6788046 | *TSC22D2* |
| ENSG00000235217 | 0.00643 | down | -2.4494698 | *TSPY26P* |
| ENSG00000167614 | 0.02553 | down | -1.7809062 | *TTYH1* |
| ENSG00000178462 | 0.00318 | down | -1.5965533 | *TUBAL3* |
| ENSG00000143367 | 0.00071 | down | -1.6977844 | *TUFT1* |
| ENSG00000122691 | 0.02343 | down | -2.2285817 | *TWIST1* |
| ENSG00000185262 | 0.01291 | down | -1.6225327 | *UBALD2* |
| ENSG00000182247 | 0.01007 | down | -1.6891682 | *UBE2E2* |
| ENSG00000168246 | 0.00010 | down | -2.0404363 | *UBTD2* |
| ENSG00000154277 | 0.00007 | down | -2.0044427 | *UCHL1* |
| ENSG00000109814 | 0.00020 | down | -2.2611563 | *UGDH* |
| ENSG00000111981 | 0.00034 | down | -1.5145181 | *ULBP1* |
| ENSG00000131015 | 0.00001 | down | -2.0147028 | *ULBP2* |
| ENSG00000131019 | 0.00026 | down | -1.9786077 | *ULBP3* |
| ENSG00000177169 | 0.00272 | down | -1.7633945 | *ULK1* |
| ENSG00000261279 | 0.03384 | down | -1.5898545 | *ULK4P1* |
| ENSG00000178081 | 0.01685 | down | -1.795748 | *ULK4P3* |
| ENSG00000255562 | 0.00000 | down | -3.149463 | *UNC93B6* |
| ENSG00000145390 | 0.00012 | down | -2.4262512 | *USP53* |
| ENSG00000143494 | 0.01619 | down | -2.3630567 | *VASH2* |
| ENSG00000205642 | 0.00555 | down | -1.6779042 | *VCX3B* |
| ENSG00000129864 | 0.00134 | down | -2.0977743 | *VCY* |
| ENSG00000129862 | 0.00125 | down | -2.1018913 | *VCY1B* |
| ENSG00000128564 | 0.00725 | down | -2.5401561 | *VGF* |
| ENSG00000206538 | 0.00422 | down | -1.7409295 | *VGLL3* |
| ENSG00000133980 | 0.00011 | down | -2.3962038 | *VRTN* |
| ENSG00000101842 | 0.00012 | down | -4.428132 | *VSIG1* |
| ENSG00000119614 | 0.00191 | down | -2.1153712 | *VSX2* |
| ENSG00000110002 | 0.00005 | down | -1.649282 | *VWA5A* |
| ENSG00000204396 | 0.00805 | down | -1.6231372 | *VWA7* |
| ENSG00000126562 | 0.00083 | down | -2.4098125 | *WNK4* |
| ENSG00000143816 | 0.00004 | down | -5.37306 | *WNT9A* |
| ENSG00000142279 | 0.03119 | down | -1.5590786 | *WTIP* |
| ENSG00000047644 | 0.01338 | down | -1.8001297 | *WWC3* |
| ENSG00000018408 | 0.01236 | down | -1.5851002 | *WWTR1* |
| ENSG00000241313 | 0.00089 | down | -2.2351365 | *WWTR1-AS1* |
| ENSG00000124343 | 0.00327 | down | -4.881592 | *XG* |
| ENSG00000252202 | 0.00298 | down | -2.5016558 | *Y_RNA* |
| ENSG00000137693 | 0.00041 | down | -1.6665753 | *YAP1* |
| ENSG00000175155 | 0.01268 | down | -1.8484318 | *YPEL2* |
| ENSG00000090238 | 0.01221 | down | -1.765887 | *YPEL3* |
| ENSG00000146839 | 0.03624 | down | -2.9877973 | *ZAN* |
| ENSG00000189167 | 0.03086 | down | -2.4774926 | *ZAR1L* |
| ENSG00000169155 | 0.01152 | down | -1.5495272 | *ZBTB43* |
| ENSG00000130584 | 0.00008 | down | -1.6400977 | *ZBTB46* |
| ENSG00000119703 | 0.03052 | down | -3.7379587 | *ZC2HC1C* |
| ENSG00000188818 | 0.00178 | down | -1.6420361 | *ZDHHC11* |
| ENSG00000206077 | 0.01505 | down | -2.7760537 | *ZDHHC11B* |
| ENSG00000196867 | 0.04621 | down | -1.5090196 | *ZFP28* |
| ENSG00000128016 | 0.00112 | down | -1.736956 | *ZFP36* |
| ENSG00000159733 | 0.00536 | down | -2.80502 | *ZFYVE28* |
| ENSG00000149050 | 0.00140 | down | -4.4783807 | *ZNF214* |
| ENSG00000138311 | 0.00279 | down | -2.9173295 | *ZNF365* |
| ENSG00000204947 | 0.01154 | down | -1.6246351 | *ZNF425* |
| ENSG00000256087 | 0.00718 | down | -1.5651025 | *ZNF432* |
| ENSG00000167785 | 0.00153 | down | -1.8644476 | *ZNF558* |
| ENSG00000198453 | 0.00470 | down | -1.5791483 | *ZNF568* |
| ENSG00000198046 | 0.00152 | down | -1.5568571 | *ZNF667* |
| ENSG00000183779 | 0.00054 | down | -3.2794547 | *ZNF703* |
| ENSG00000214534 | 0.00553 | down | -1.9882946 | *ZNF705E* |
| ENSG00000152475 | 0.01468 | down | -1.7875766 | *ZNF837* |
| ENSG00000221923 | 0.04947 | down | -2.2158146 | *ZNF880* |
| ENSG00000132003 | 0.00004 | down | -1.6764138 | *ZSWIM4* |
| ENSG00000130449 | 0.01221 | down | -1.571306 | *ZSWIM6* |
| ENSG00000214655 | 0.00167 | down | -1.5368632 | *ZSWIM8* |

**Suppl. Table 2.**
