# Supplementary material for: Reduced miR-26b Expression in Megakaryocytes and Platelets Contributes to Elevated Level of Platelet Activation Status in Sepsis
Source: Int J Mol Sci. 2020 Jan 29;21(3):866. doi: 10.3390/ijms21030866 (PMC7036890; doi:10.3390/ijms21030866)
Supplement: Supplementary file 1 [file ijms-21-00866-s001.zip › ijms-696391-supp-final/Suppl.Table3_Szilagyi_final_mod.docx]

| **miRNA** | **Stem-loop primers for reverse transcription** | **Forward primers for**  **RT-qPCR** | **Universal reverse primer for**  **RT-qPCR** | **Universal Probe Library probe #21** | **Mature miRNA sequences** |
| --- | --- | --- | --- | --- | --- |
| hsa-miR-26b-5p | 5' - GTTGGCTCTGGTGCAGGGTCCGAGGTATTCGCACCAGAGCCAAC ACCTAT - 3' | 5' - GTTTGGGTTCAAGTAATTCAGG - 3' | 5' - GTGCAGGGTCCGAGGT - 3' | 5' - TGGCTCTG - 3' | 5' - UUCAAGUAAUUCAGGAUAGGU - 3' |
| hsa-miR-451 (5’) | 5' -GTTGGCTCTGGTGCAGGGTCCGAGGTATTCGCACCAGAGCCAAC AACTCA - 3' | 5' - GTTTGGAAACCGTTACCATTAC - 3' | 5' - GTGCAGGGTCCGAGGT - 3' | 5' - TGGCTCTG - 3' | 5' - AAACCGUUACCAUUACUGAGUU - 3' |
| hsa-RNU-43 | 5' - GTTGGCTCTGGTGCAGGGTCCGAGGTATTCGCACCAGAGCCAAC AATCAG - 3' | 5' - GTGAACTTATTGACGGGCG - 3' | 5' - GTGCAGGGTCCGAGGT - 3' | 5' - TGGCTCTG - 3' | 5' - GAACUUAUUGACGGGCGGACAGAAACUGUGUGCUGAUUGUCACGUUCUGAUU - 3' |
| hsa-miR-223-3p | 5' - GTTGGCTCTGGTGCAGGGTCCGAGGTATTCGCACCAGAGCCAAC TGGGGT - 3' | 5' - GTTGGGTGTCAGTTTGTCAAAT - 3' | 5' - GTGCAGGGTCCGAGGT - 3' | 5' - TGGCTCTG - 3' | 5' - UGUCAGUUUGUCAAAUACCCCA - 3' |

| **mRNA** | **Forward primers for RT-qPCR** | **Reverse primers for RT-qPCR** | **Amplicon lenght** |
| --- | --- | --- | --- |
| SELP | 5' - CCATTGTCTAGAGGGCCAGT - 3' | 5' - GGGCTTCCTGGATAGTCAATG - 3' | 119 nt |
| IL1B | 5' - AGCCAGGACAGTCAGCTCTC - 3' | 5' - AGAGGCCTGGCTCAACAA - 3' | 65 nt |
| DICER | 5' - TGTTCCAGGAAGACCAGGTT - 3' | 5' - ACTATCCCTCAAACACTCTGGAA - 3' | 76 nt |
| ITGA2B | 5' - AGCCTCAATGTGTCCCTACC - 3' | 5' - AGTCCAGGACGATTCGTGTC - 3' | 103 nt |
| RPLP0 (36B4) | 5' - ATGCAGCAGATCCGCATGT- 3' | 5' - TCATGGTGTTCTTGCCCATCA - 3' | 64 nt |

**Suppl. Table 3.**
