# Supplementary figures and images for: Reduced miR-26b Expression in Megakaryocytes and Platelets Contributes to Elevated Level of Platelet Activation Status in Sepsis
Source: Int J Mol Sci. 2020 Jan 29;21(3):866. doi: 10.3390/ijms21030866 (PMC7036890; doi:10.3390/ijms21030866)

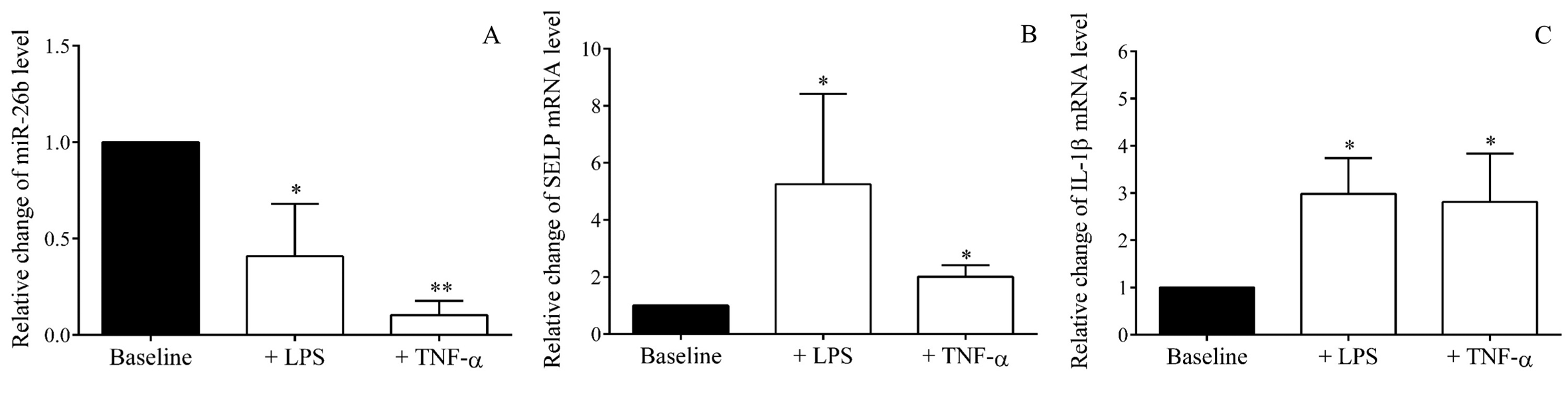

Supplement: Supplementary file 1 [file ijms-21-00866-s001.zip › ijms-696391-supp-final/Suppl.Fig.1_Szilagyi_final.tif]

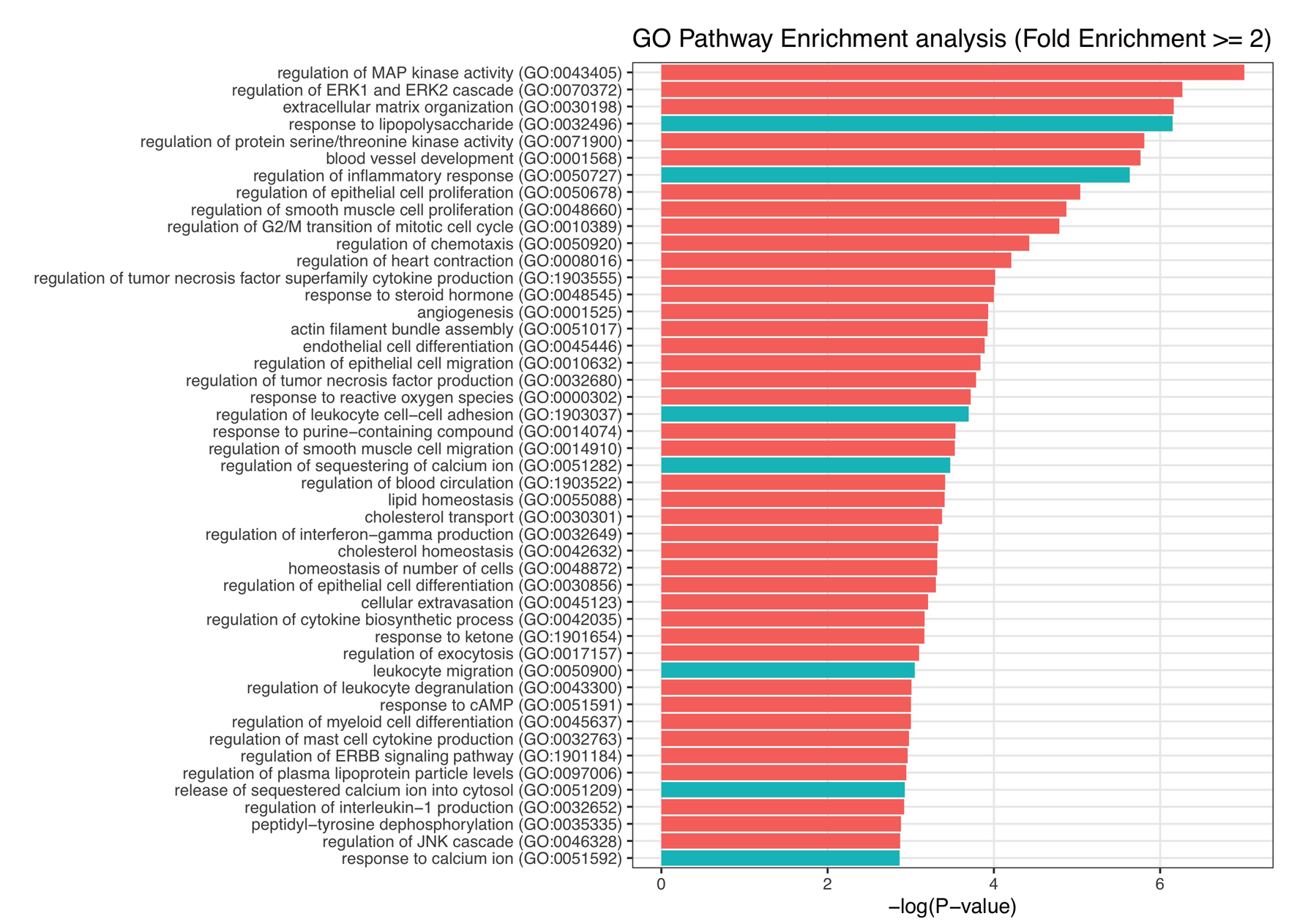

Supplement: Supplementary file 1 [file ijms-21-00866-s001.zip › ijms-696391-supp-final/Suppl.Fig.2_Szilagyi_final.tif]
